# Morphological variability of *Carex buekii* (Cyperaceae) as a function of soil conditions: a case study of the Central European populations

**DOI:** 10.1038/s41598-022-15894-0

**Published:** 2022-07-11

**Authors:** Helena Więcław, Beata Bosiacka, Richard Hrivnák, Zygmunt Dajdok, Attila Mesterházy, Jacob Koopman

**Affiliations:** 1 Institute of Marine and Environmental Sciences, University of Szczecin, Adama Mickiewicza 18, 70383 Szczecin, Poland; 2 Institute of Botany, Plant Science and Biodiversity Center, Slovak Academy of Sciences, Dúbravská cesta 9, 845 23 Bratislava, Slovakia; 3 Department of Botany, Faculty of Biological Sciences, University of Wrocław, Kanonia 6/8, 50328 Wrocław, Poland; 4 Centre for Ecological Research, Wetland Ecology Research Group, Bem tér 18/C, 4026 Debrecen, Hungary; 5 Choszczno, Poland

**Keywords:** Ecology, Plant sciences

## Abstract

*Carex buekii* is a highly adaptive species showing a fairly wide ecological spectrum. It belongs to the group of river corridor plants which are vulnerable to any human activity directed at transformation of river valley habitats worldwide. This study was aimed at: determining the phenotypic variability of the species in the central part of its range, examining effects of soil conditions on the sedge’s morphological traits, and finding out whether the phenotypic plasticity observed may have taxonomic implications. A total of 487 specimens from 26 populations were collected in Hungary, Poland and Slovakia and tested by univariate, bivariate, and multivariate statistical methods. The analysis involved 16 morphological traits and 7 soil parameters (organic matter, pH, potassium, phosphorus, nitrogen, magnesium, calcium). Soil conditions were shown to affect the *C*. *buekii* morphology; particularly important was potassium, the only soil parameter that was indicated as a factor affecting intra-specific variability. Sites with lower contents of bioavailable potassium hosted *C*. *buekii* individuals which were generally smaller than those at sites showing higher soil potassium contents. The relationship held true also with respect to generative traits important in sedge taxonomy, i.e. utricle and beak lengths. Consideration of morphological differences only, without analysing relationships between morphology and soil conditions, could have resulted in distinguishing new entities at the level of species, subspecies or variety. Thus, knowledge on the range of phenotypic plasticity in field populations seems to be of a key importance in taxonomic studies.

## Introduction

Diversity of soil conditions may affect the morphology of plants growing in heterogeneous habitats^1,2^. Usually, the culm height, number of leaves, leaf surface area, and biomass were observed to increase with increasing nutrient contents^3,4^. Phenotypic variability associated with habitat conditions may lead to a distinct intra-specific differentiation between morphotypes, and even to separation of new taxa^5^. On one hand, the morphological traits used to identify and describe organisms are major practical criteria in plant taxonomy, as numerous taxonomic descriptions are based on morphological data^6^. On the other hand, using morphological traits alone for taxon delimitation, with no consideration for its plasticity, may lead to misidentifications^5^. Plasticity is considered to be a major source of phenotypic variation as it affects natural selection and, consequently, patterns of diversification among populations and species^7^.

*Carex buekii* Wimm., as a highly adaptive species of a fairly wide ecological spectrum, is a suitable model species for the research on phenotypic variability against ecological background. This sedge from the section *Phacocystis* Dumort. occurs in central-eastern Europe, in the northern part of the Balkan Peninsula and in south-eastern Asia^8^. *C. buekii* is a perennial plant more than 100 cm tall, with long and thick rhizomes. The species is distinguished based on, *inter alia*, dark reddish-brown basal leaf sheaths which display a characteristic reticulate-fibrous structure, the shiny upper side of the broad leaves (more than 1 cm) and nerveless or indistinctly nerved utricles with very short beaks^9^.

*C. buekii* is associated mainly with wetlands in river valleys and belongs to the so-called river corridor plants^10^. It is a group of species vulnerable to any human activity directed at transformation of river valley habitats worldwide. *C. buekii* grows both on river floodplains and in areas located at a considerable distance away from the river, including man-made habitats such as ditch and canal banks, bridgeheads and river embankments as well as roadsides. It usually occurs in nutrient-rich habitats, but is also capable of colonising relatively nutrient-poor ones; it grows on both acidic and alkaline soils (pH 3.3–7.4) with diverse concentrations of assimilable elements^11^.

Floodplains are relatively nutrient-rich^10,12^, particularly in valleys of large rivers, with a higher mineralisation rate and higher amounts of available macroelements^13,14^. On central-European plains, the corridors of large rivers are covered mainly by clay-rich Holocene deposits, surrounded by predominantly sandy material^10^. In addition, regular flooding of river valleys leads to distinct differences in nutrient contents, with floodplains representing relatively nutrient-rich corridors in a nutrient-poor landscape^15^.

The present study was aimed at: (1) determining the phenotypic variability of *C*. *buekii* in the central part of its range, (2) examining effects of soil conditions on the sedge’s morphological traits, and (3) finding out whether the observed phenotypic plasticity may have taxonomic implications.

## Material and methods

### Field studies and specimen collection

**Figure 1:**
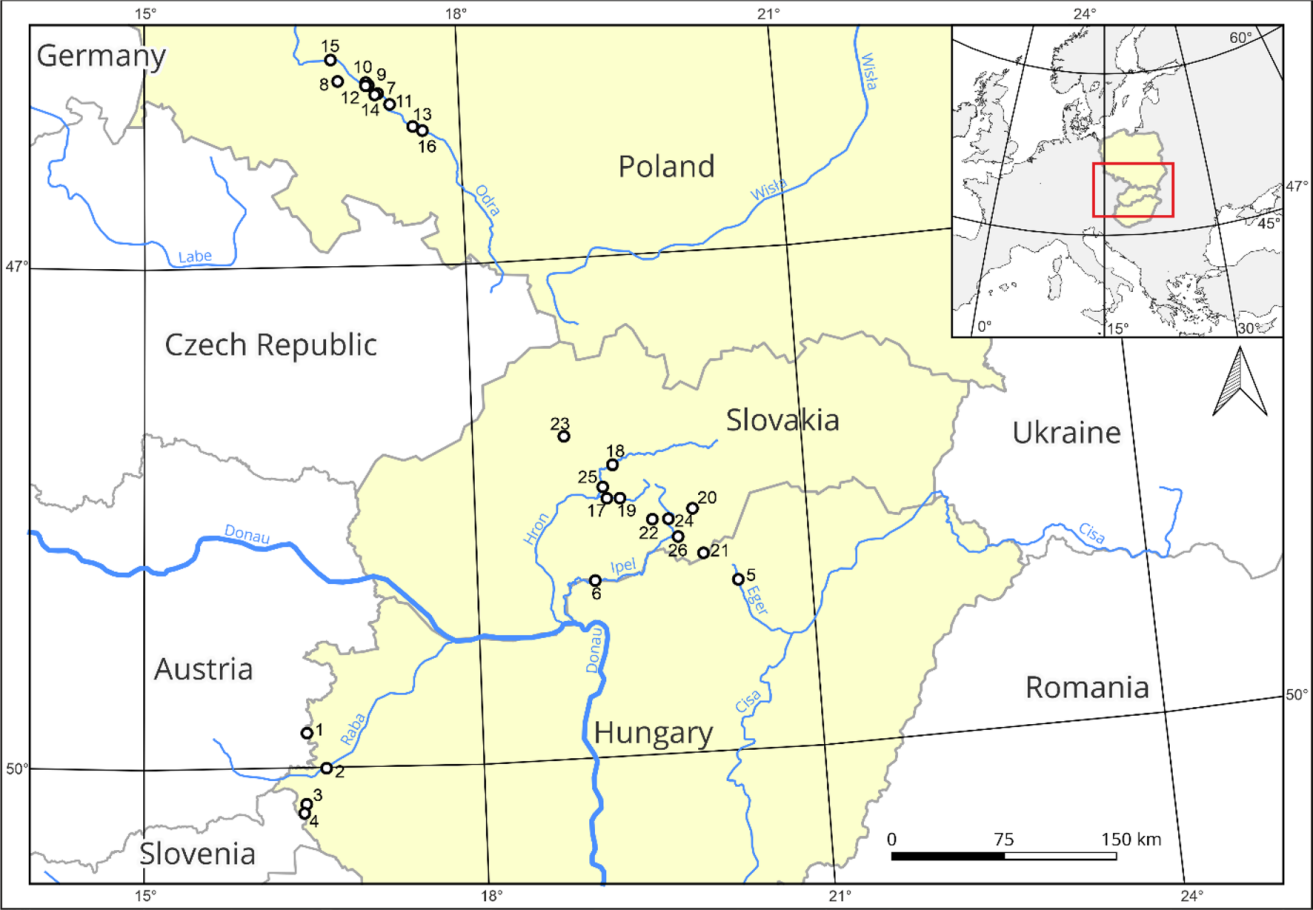
Location of *Carex buekii* collection sites in Poland, Slovakia and Hungary. The map was made in QGIS version 3.22 available at https://qgis.org.

A total of 487 specimens from 26 wild populations (10–29 specimens per population, depending on the population size) of *C*. *buekii* in three Central European countries were examined. Field studies were conducted in Poland (119 specimens from 10 populations), Slovakia (262 specimens from 10 populations), and Hungary (106 specimens from 6 populations) (Fig. 1; Table S1). Specimens from a population were collected 3–6 m apart from one another to reduce the chance of collecting individuals from the same clone. The formal identification of the plant material was carried out by H. Więcław and J. Koopman. Voucher specimens for each population were deposited in the publicly available Herbarium Stetinensis at the University of Szczecin (SZUB)^16^.

No official permits for our research were required because (i) all field research was conducted outside protected areas, at sites where sedges were abundant, (ii) the study did not concern protected taxa (Zákon/the Law No. 15/2005 Z.z. and Vyhláška/the Edict No. 24/2003 Z.z., Regulation of the Minister of the Environment of 2014, item 1409). Only a negligible part of each *Carex buekii* population was collected (always without root systems); therefore, no negative effects on the population were induced.

### Morphological traits and measurements

A total of 16 morphological traits were determined (Table 1). Utricles, beaks and glumes were measured to 0.01 mm under a stereomicroscope. Five utricles and glumes, each from the middle part of a spike of each specimen, were isolated for measuring and the results were averaged. Other measurements were taken with a vernier calliper (to 0.05 cm: the spike size, widths of bracts and leaves, the peduncle length) and a ruler (to 0.1 cm: the culm height, leaf and bract lengths). The culm height was measured from the soil level to the top of the uppermost male spike. For each specimen, the length and width of the uppermost cauline leaf were measured. The leaf width was measured in the leaf’s central part.

**Table 1:** Quantitative traits used in morphological analyses.

No.	Traits	Abbreviations
1	Culm height (cm)	CH
2	Cauline leaf length (cm)	CLL
3	Cauline leaf width (cm)	CLW
4	Number of female spikes (no)	NFS
5	Number of male spikes (no)	NMS
6	Inflorescence length (cm)	IL
7	Male spike length (cm)	MSL
8	Male spike width (cm)	MSW
9	Female spike length (cm)	FSL
10	Female spike width (cm)	FSW
11	Peduncle length of the lowest female spike (cm)	PL
12	Lowest bract length (cm)	BL
13	Utricle length (mm)	UL
14	Utricle beak length (mm)	UBL
15	Ratio of beak length to urticle length (%)	UBL/UL
16	Glume length (mm)	GL

### Soil analysis

Soil samples were collected at each site, from the depth of 0–25 cm, with Egner's soil sampler. At each site, the samples were collected from three spots, the samples being subsequently combined into one to be used in laboratory assays. The soil samples were dried at room temperature and then rubbed through a sieve to remove fractions larger than 1 mm. The soil material prepared this way was used to determine the organic matter content (as a loss on ignition at 550 °C), pH (potentiometrically, in 1 M KCl), contents of assimilable nutrients: phosphorus (P) and potassium (K) using the Egner-Riehm method, magnesium (Mg) using Schachtschabel’s method, calcium (Ca) using atomic absorption spectrophotometry, and total nitrogen (N) using the Kjeldahl method (follwing the American Society of Agronomy^17^).

### Data processing

Significance of differences between the data distribution and the theoretical normal distribution was examined using the Shapiro–Wilk test. As the distributions of most data sets deviated from normal, the non-parametric Mann–Whitney U test, Kruskal–Wallis test and Dunn’s multiple comparisons test were used to test for significance of differences between *C*. *buekii* populations. Relationships between the morphological traits and soil properties were examined with Spearman’s rank association test.

A preliminary sorting of the specimens was carried out using the Euclidean distance-based Ward’s minimum variance. Subsequently, the principal component analysis (PCA, on the correlation matrix) based on the complete data set, comprising all morphological traits, was carried out to quantify *C*. *buekii*’s morphological variability. The data used in the multivariate analyses were standardised so that each variable would have a mean of 0 and a standard deviation of 1. All the analyses were run in Statistica v. 13.1 for Windows^18^.

Sample distribution patterns and morphological traits in relation to soil variables were analysed by the redundancy analysis (RDA) using CANOCO v. 4.51^19^. The Monte Carlo permutation test was applied to determine statistical significance of soil properties applicable to explaining the *C*. *buekii* variability.

## Results

### Variability of morphological traits and its relationship with soil properties

Biometric analyses showed the most variable traits in *C*. *buekii* to include the peduncle length, PL (*V* = 63%); the culm height, CH; the bract length, BL and the number of male spikes, NMS (*V* ≥ 30%); the utricle beak length, UBL; the female spike length, FSL; and the inflorescence length, IL (*V* ≥ 25%) (Table 2). The mean plant height of 117.20 cm was associated with a standard deviation of 23.84 cm, indicating a relatively high degree of data dispersion. A high data variability was also observed in the cauline leaf length (13.08 cm and 40.04 cm standard deviation and mean, respectively), bract length (5.23 cm and 16.31 cm standard deviation and mean, respectively), and inflorescence length (4.67 cm and 18.57 cm standard deviation and mean, respectively). The variation amplitudes of the utricle length, UL and glume length, GL were narrow (coefficients of variation lower than 15%), indicating a low phenotypic plasticity with respect to these relatively homogeneous characters (Table 2).

**Table 2:** Morphological traits of *Carex buekii*.

Traits	Mean ± *SD*	Median	Min	Max	*IQR*	*V* [%]
CH [cm]	117.25 ± 23.84	119.8	33.50	193.20	15.15	20.34
CLL [cm]	40.04 ± 13.08	38.00	13.60	77.50	9.7	32.68
CLW [cm]	0.55 ± 0.13	0.55	0.22	0.98	0.09	23.28
NFS [no]	3.56 ± 0.66	4.00	2.00	6.00	0.50	18.62
NMS [no]	2.31 ± 0.73	2.00	1.00	6.00	0.50	31.56
IL [cm]	18.57 ± 4.67	18.20	7.60	39.40	2.95	25.17
MSL [cm]	4.17 ± 1.01	4.10	1.20	9.90	0.55	24.24
MSW [cm]	0.33 ± 0.06	0.32	0.18	0.51	0.04	16.77
FSL [cm]	7.04 ± 1.96	6.90	1.40	12.60	1.30	27.82
FSW [cm]	0.36 ± 0.06	0.35	0.22	0.59	0.50	16.01
PL [cm]	1.97 ± 1.24	1.70	0.20	8.70	0.70	62.73
BL [cm]	16.31 ± 5.23	15.80	3.10	38.10	3.25	32.05
UL [mm]	2.29 ± 0.33	2.28	1.52	3.55	0.21	14.54
UBL [mm]	0.26 ± 0.07	0.24	0.13	0.90	0.04	27.70
UBL/UL	0.11 ± 0.03	0.11	0.06	0.45	0.01	23.28
GL [mm]	2.33 ± 0.30	2.34	1.58	3.12	0.23	12.84

*SD,* standard deviation; Min, minimum value; Max, maximum value; *IQR,* interquartile range; *V,* coefficient of variation.

Variability of the *C*. *buekii* morphological traits was found to be associated with soil conditions. Spearman’s rank association test showed significant (*p* ≤ 0.05) positive correlations between (1) the soil contents of potassium and the culm height, CH (*r*_*s*_ = 0.635); the cauline leaf length, CLL (*r*_*s*_ = 0.447); the cauline leaf width, CLW (*r*_*s*_ = 0.643); and the bract length, BL (*r*_*s*_ = 0.448); (2) the soil pH and the cauline leaf width, CLW (*r*_*s*_ = 0.433) and the male spike length, MSL (*r*_*s*_ = 0.465); (3) the soil contents of phosphorus and the cauline leaf width, CLW (*r*_*s*_ = 0.432) and the bract length BL (*r*_*s*_ = 0.448). Significant negative correlations were observed between (1) the soil contents of magnesium and the number of female spikes, NFS (*r*_*s*_ = −0.401) and the peduncle length, PL (*r*_*s*_ = −0.394); (2) the soil contents of calcium and the peduncle length, PL (*r*_*s*_ = −0.418) (Table S2).

### Between-populations variability and its relationship with soil properties

The non-parametric Kruskal–Wallis test detected significant differences between *C*. *buekii* populations (*p* ≤ 0.05) in all the morphological traits analysed. The post-hoc Dunn's multiple comparisons test identified the largest differences in morphological traits between the following population pairs: 2 vs 18, 3 vs 12, and 3 vs 21, with significant differences in 10 traits out of 16, and 1 vs 23, 2 vs 12, 2 vs 15, 2 vs 23, 19 vs 23, and 20 vs 23, with significant differences in 9 traits (Table S3).

**Figure 2:**
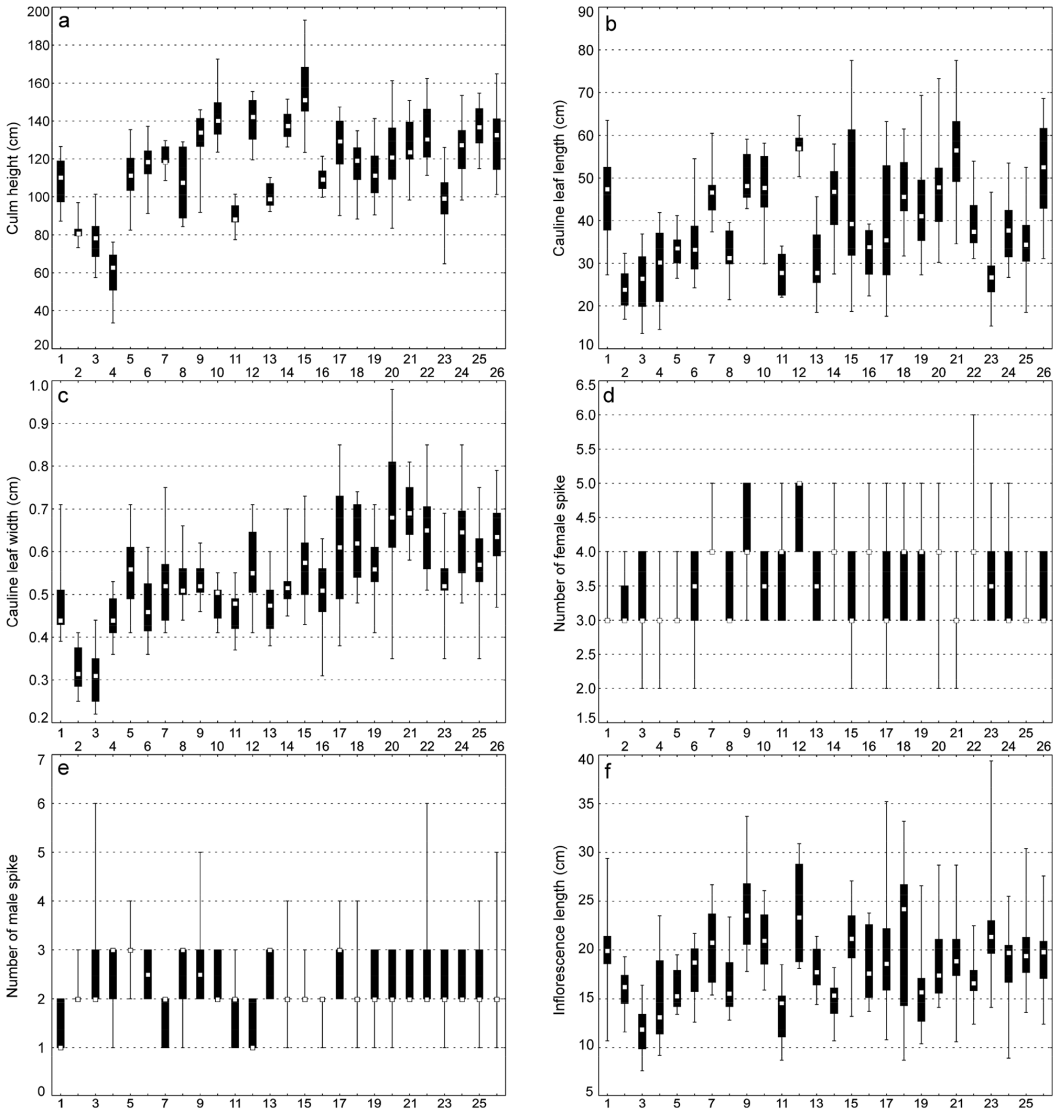

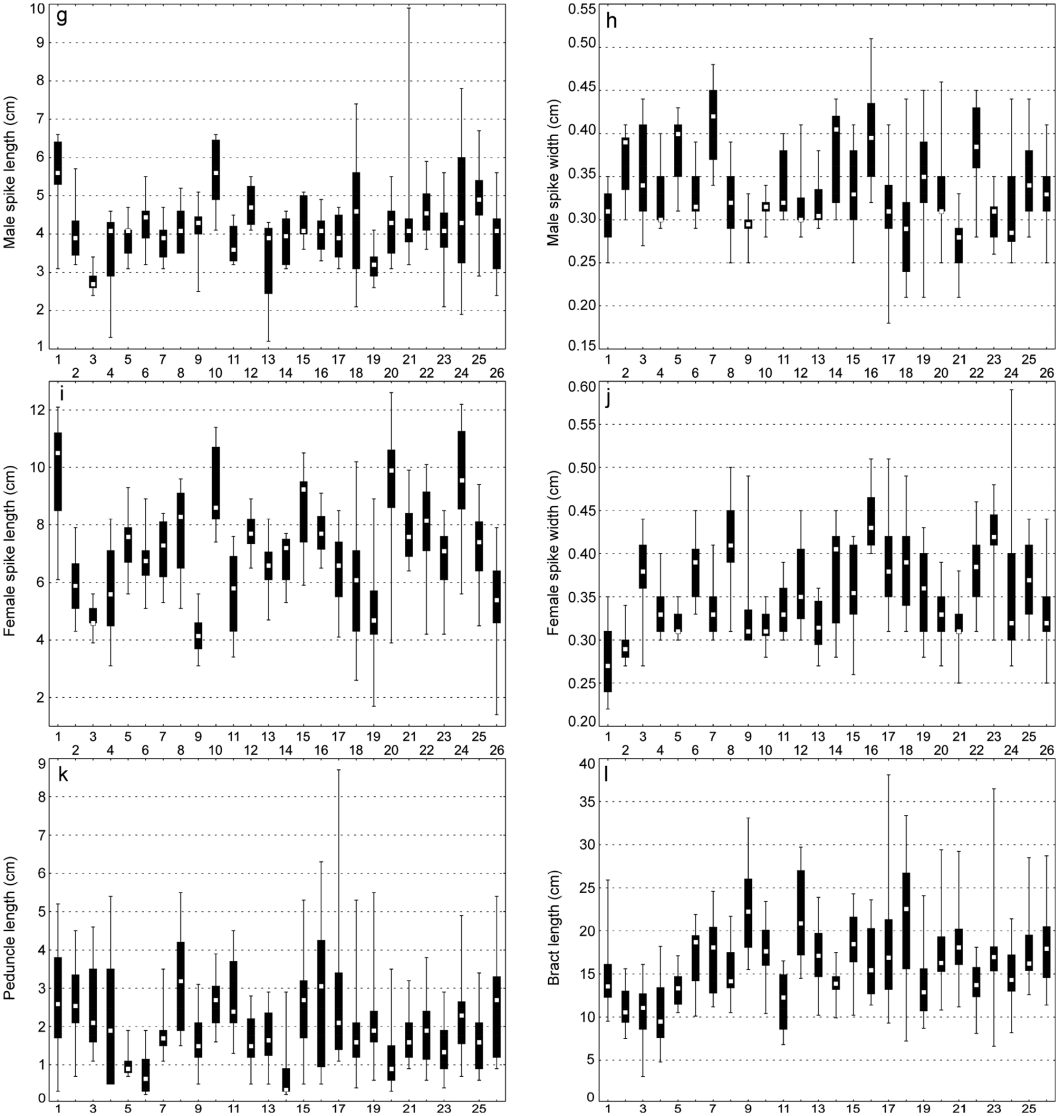

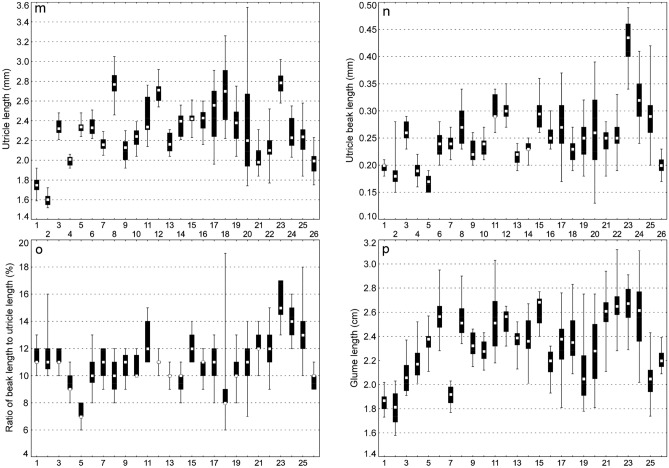
Ranges of morphological traits of *Carex buekii* populations. Large boxes indicate 25–75% of the interquartile ranges; small boxes represent medians; 1, 2, 3 ….26 are population numbers. The populations are numbered as in Table S1.

In most traits, the ranges of values were overlapping between the populations (Fig. 2). Generally, the most distinct and significant differences were observed in the culm height (CH) of populations 4 and 15; the leaf length (CLL) of populations 12 and 2; the leaf width (CLW) of populations 2, 3 and 20; the male spike length (MSL) of populations 3 and 10; the female spike length (FSL) of populations 1 and 9; the female spike width (FSW) of populations 1 and 16; the utricle length (UL) of populations 1, 2 and 8, 12, 23; the beak length (UBL) of populations 1, 2, 4, 5 and 23; and the glume length (GL) of populations 1, 2, 7 and 15 (Fig. 2). The mean value of the beak length to utricle length ratio (UBL/UL) was at its lowest and highest in populations 5 and 23, respectively.

**Figure 3:**
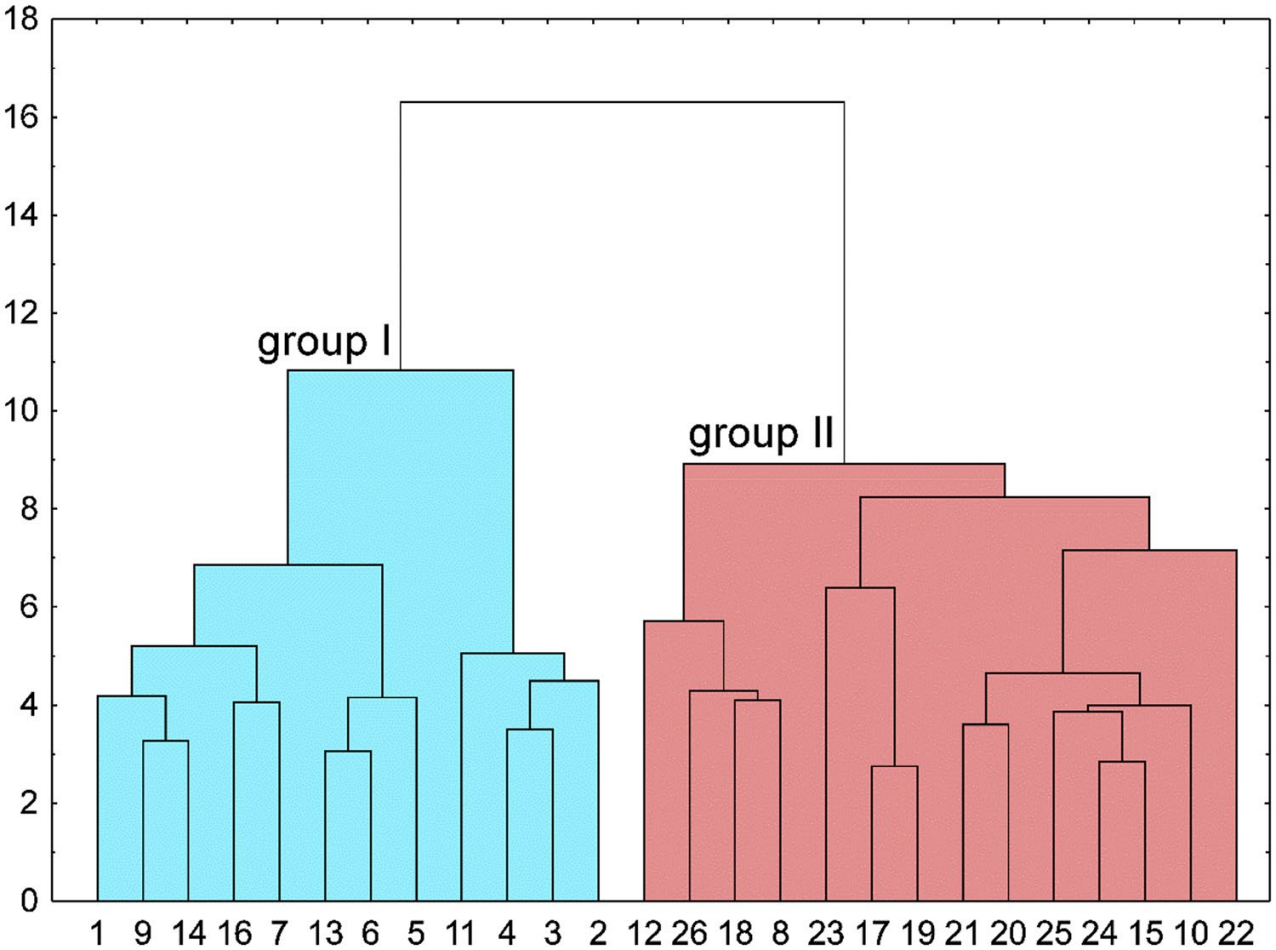
Results of Euclidean distance-based cluster analysis (Ward’s minimum variance) for *Carex buekii* populations. The populations are numbered as in Table S1.

**Figure 4:**
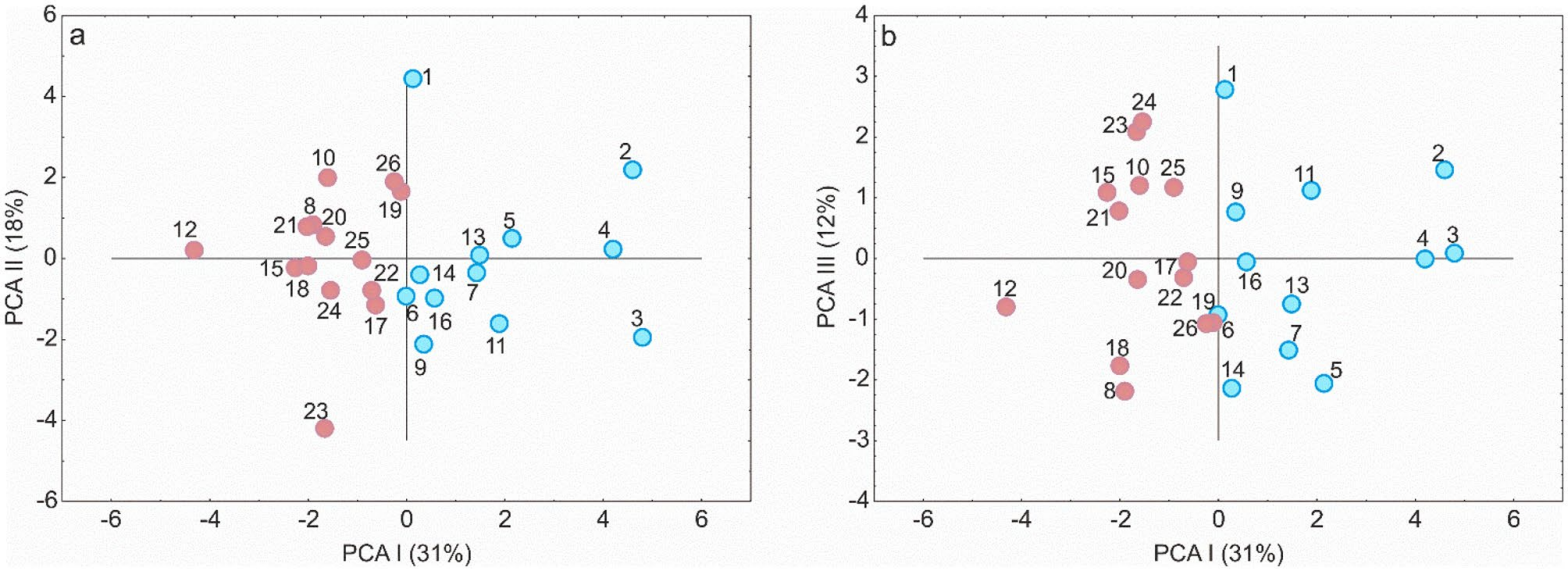
Distribution of the *Carex buekii* populations studied in two-dimensional space (Principal Component Analysis, PCA). Blue and pink circles represent group I and group II populations, respectively (cf. Fig. 3). The populations are numbered as in Table S1. Loadings for the first axis (PC1) (only absolute values ≥ 0.5): CH (culm height) =  − 0.82; CLL (cauline leaf length) =  −0.73; CLW (cauline leaf width) = −0.72; IL (inflorescence length) =  −0.83; MSL (male spike length) =  −0.61; BL (bract length) =  − 0.85; and GL (glume length) =  −0.51. Loadings for the second axis (PC2) (only absolute values ≥ 0.5): FSW (female spike width) =  −0.80; UL (utricle length) =  −0.75; UBL (utricle beak length) =  −0.69; and GL (glume length) =  −0.59. Loadings for the third axis (PC3) (only absolute values ≥ 0.5): FSL (female spike length) = 0.51; PL (peduncle length) = 0.50; and UBL/UL (beak length to utricle length ratio) = 0.70.

Based on the cluster analysis involving 16 morphological traits, the *C*. *buekii* specimens were assigned to two different groups (Fig. 3). This division reflects the distribution of the samples (populations) in the phenetic space (Fig. 4). Populations representing group I (8, 10, 12, 15, 17, 18, 19, 20, 21, 22, 23, 24, 25, 26) occupy the space on the left-hand side of the first (horizontal) axis, whereas populations making up group II (1, 2, 3, 4, 5, 6, 7, 9, 11, 13, 14, 16) are placed in the right-hand side of the plot (Fig. 4). Along the first axis, populations 2, 3 and 4 (right-hand side) as well as population 12 (left-hand side) are farthest away from the plot centre. The strongest effect on the first PCA axis was exerted by the culm height (CH), the cauline leaf length and width (CLL and CLW), the inflorescence length (IL), the male spike length (MSL), the bract length (BL), and the glume length (GL). The second axis was determined mostly by the female spike width (FSW), the utricle length (UL), the utricle beak length (UBL), and the glume length (GL) (Fig. 4A). The traits associated with the third axis included primarily those related to the female spike length (FSL), the peduncle length (PL), and the beak length to utricle length ratio (UBL/UL) (Fig. 4B). The first three principal component axes, taken together, explained 61% of the variance (31%, 18% and 12%, respectively). The fourth principal component axis explained 10% of the variance, other axes explaining a still lower percentage (Table S4).

The Mann–Whitney U test showed the two *C*. *buekii* groups described above to differ significantly in 13 morphological traits (Table S5). The group I specimens are generally smaller, show shorter and narrower leaves, have fewer female spikes, their inflorescences are shorter, their male spikes are shorter and wider, their female spikes are narrower, their bracts are shorter, their utricles are smaller, and the utricle beaks are shorter than those in group II. In addition, the beak length to utricle length ratio in group I is lower than that in group II (Fig. S1). Group I contains Hungarian and some Polish populations, whereas group II includes Slovak populations and the remaining Polish ones (Table S1).

**Figure 5:**
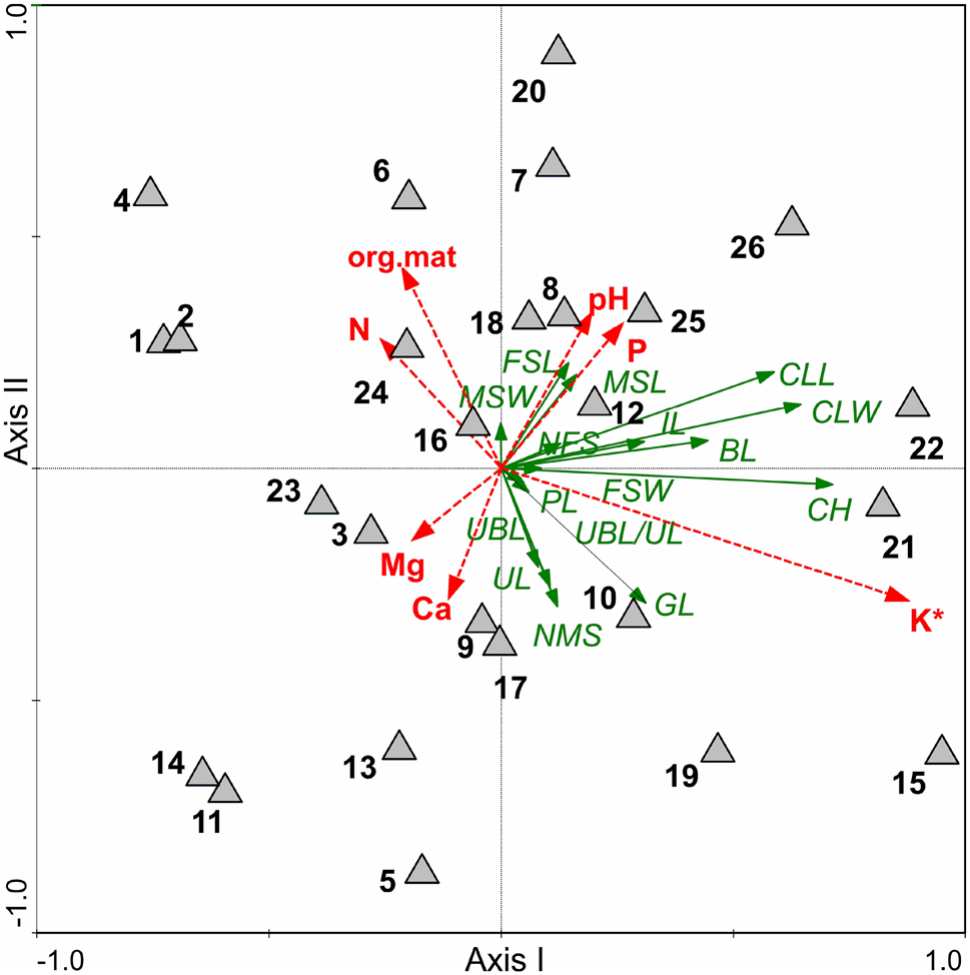
Ordination plot of populations (triangle), morphological traits and soil properties along the first two RDA axes; org. mat., organic matter content; pH, soil pH; N, nitrogen; P, phosphorus; K, potassium; Ca, calcium; *variables statistically significant (p ≤ 0.05). For trait codes see Table 2. The populations are numbered as in Table S1.

Results of the redundancy analysis (RDA) showed all the variables used to account for 48.2% of the total variance in the data (Table 3). Results of the step-wise forward selection of variables demonstrated the soil potassium content to be the only significant variable (Table 4). The *C*. *buekii* populations were scattered in the ordination space. The location of populations 15, 19, 21, 22 and 26 was associated with a relatively high soil potassium content as well as low nitrogen and organic matter contents (Fig. 5). Those populations were composed of relatively tall individuals with long and wide leaves. In contrast, populations 1, 2 and 4, which grew in relatively potassium-poor soils, consisted of specimens usually showing lower values of many morphological traits, including the taxonomically significant generative ones such as the utricle and beak lengths (Figs. 2 and 5; Table S1).

**Table 3:** Summary of redundancy analysis (RDA) of samples collected at *Carex buekii* sites in Poland, Slovakia, and Hungary.

Axes	I	II	III	IV
Eigenvalues	0.468	0.008	0.005	0.000
Species-environment correlations	0.718	0.352	0.551	0.342
Cumulative percentage variance of species data	46.8	47.6	48.1	48.2
Cumulative percentage variance of species–environment relation	97.1	98.8	99.9	99.9
Sum of all eigenvalues/Total inertia	1.00			
Sum of all canonical eigenvalues	0.482			
Percentage of explained species data variance	48.2

**Table 4:** Forward selection results with test of variable significance for samples collected at *Carex buekii* sites in Poland, Slovakia, and Hungary.

Soil properties	Lambda A	Explained data variance [%]	*F*-ratio	*p*-value
K*	0.36	36.0	13.43	0.002
Ca	0.04	4.0	1.53	0.220
P	0.04	4.0	1.45	0.266
pH	0.01	1.0	0.57	0.500
N	0.01	1.0	0.52	0.538
Mg	0.02	2.0	0.41	0.600
org.mat	0.00	0.0	0.20	0.796

*Variables statistically significant (p ≤ 0.05).

## Discussion

### Effects of potassium on plant growth and morphology

The potassium content was significantly correlated with the magnitude of some morphological traits of *C*. *buekii*. It was the only soil-related variable, among those analysed, to be significant, as determined by the RDA analysis. Doubtless, potassium—in addition to nitrogen and phosphorus—is one of the basic elements necessary for plant growth and development^20,21^. Potassium is the most frequent inorganic cation in plants, and accounts for up to 10% of the plant dry weight^22,23^. Potassium contents in *Carex* tissues are relatively high^24^, but are highly species-specific and habitat-dependent, as they are influenced mainly by the soil water content, soil type, and potassium availability^25,26^.

Changes in water level in flooding-prone areas usually constitute a major stressor which affects marsh vegetation growth and physiological processes^27^. As a rule, soil flooding limits plant growth by reducing oxygen penetration to the roots^28^. Some marsh plants are capable of diminishing damage due to oxygen deficiency and can increase their ability to tolerate flooding by biomass relocation (more biomass in the above-ground part to increase oxygen availability and less biomass in the below-ground part to diminish oxygen utilisation) and adaptation of shoot morphology, e.g. formation of elongated shoot organs such as internodes and petioles^29,30^. *C. buekii* seems to be relatively tolerant of disturbance due to flooding and increased water level^11^. However, similarly to other marsh species, its response to long-lasting flooding involves growth restriction (as shown by observations not used in the analyses presented in this work).

The plant growth on floodplains of large rivers, the usual *C*. *buekii* habitat, is practically not nutrient-limited. The nutrient supply there is mainly associated with water quality, flooding regime, and potential effects of agricultural use^13^. Flooding has profound impacts on the soil nutrient stoichiometry. Cao et al.^31^ found flooding to have significantly increased the contents of C and N in the terrestrial ecosystems they studied. However, the potassium form available to plants is easily leached out by the water, hence the amount of available potassium is frequently lower than that of nitrogen or phosphorus^32,33^.

*C. buekii* may grow on inundated meadows as well as on the overdried soil of dykes and embankments^11^. As shown by Sardans et al.^32^, consequences of overdrying are more important for the content of bioavailable potassium than phosphorus in the soil, most probably because potassium is more mobile in the soil and its absorption depends strongly on water transpiration and is associated with the osmotic control exerted by plants. Reduction of the soil moisture implied a decline in the soil diffusion capacity and a decreased amount of potassium available to plants^32^.

Generally, the potassium-poorer sites supported smaller *C*. *buekii* specimens, that is numerous morphological traits attained lower values compared to those recorded at potassium-richer sites. The utricle and beak lengths, traits important in sedge taxonomy, were also shorter. Effects of available potassium deficiency on plant morphology has been studied so far in cultivated species. For example, soil potassium deficiency was a cause of reduced growth of maize^34^, cotton^35^ and white clover^36^. Moreover, at a potassium deficiency, the leaf emergence rate was observed to be lower in rice^37^, tomatoes^38^ and maize^34^.

The potential of potassium to stimulate plant growth is directly related to its role in maintenance of the cellular turgor^39^ and indirectly to its role in controlling the osmotic potential of the stomata guard cells^40^; it is also associated with interactions and feedbacks between cellular potassium contents and the synthesis of abscisic acid (ABA) and auxin^39^.

In the present study, the soil potassium content was the only soil property significant for the morphological variability of *C*. *buekii.* It could have been important for the availability of other elements to the sedge. Interactions between potassium and other elements have been described in the literature; for example, high potassium concentrations in soil solutions inhibit magnesium uptake^41^; potassium deficiency may hamper absorption of calcium and sodium^42^ as well as nitrogen^43^. Reisch et al.^44^ observed effects of soil nutrient conditions on clonal diversity and genetic variation in *C*. *nigra*; both increased with the phosphorous concentration and decreased with that of potassium. Such interactions at the *C*. *buekii* sites we studied cannot be ruled out. Plant morphology is likely to be dependent on a number of factors and their interactions, and—as a rule—conforms to the primary limiting factor^45^.

### Phenotypic plasticity within the genus *Carex* and its taxonomic implications

Variability of *C*. *buekii* specimens allowed to distinguish between two morphologically different groups. However, despite the differences in generative traits important for taxonomy (mainly the utricle size and utricle beak length), we think it is not appropriate to establish new taxa at the level of species, subspecies or even a variety. The morphological variability observed is most likely a result of *C*. *buekii* adaptation to the variable habitat conditions on floodplains and at sites altered by human activities such as construction of levees and canals.

As shown by studies on the *C*. *flava* agg., periodic flooding, local desiccation, trampling, sun exposure, and local edaphic conditions may lead to the emergence of different morphotypes^5^. The type of land use (grazing, mowing) may affect plant morphology as well^46^. Sedges growing at sites with poorer light conditions employ a shade avoidance strategy and develop higher culms as an advantage in the relatively strong competition for light^47^. Abnormally developed spikes are frequently observed in dry areas, e.g. in the section *Racemosae* taxa^48^. Lower temperatures in the mountains limit cell divisions and result in a smaller size of the plant (dwarf morphotypes). Some isolated montane *Carex* populations support morphotypes so different as to merit a separate taxonomic status, e.g. *C*. *lepidocarpa* subsp. *nevadensis* in the Sierra Nevada and *C*. *lepidocarpa* subsp. *ferraria* in the High Atlas^49^.

The botanical literature contains several reports on taxa within the genus *Carex* which have lost their previous taxonomic status after their wide phenotypic plasticity, resulting from adaptation to local habitat conditions, was examined. Such taxa include *C. viridula* Michx. var. *pulchella* (Lönnr.) B. Schmid from the section *Ceratocystis* Dumort., known also as the subspecies *C*. *viridula* Michx. subsp. *pulchella* (Lönnr.) Malyschev or, earlier, as the species *C*. *pulchella* (Lönnr.) Lindm.^5,50^. Another example of a controversial taxon is *C*. *norvegica* subsp. *pusteriana* (Kalela) Á.Löve & D.Löve (sect. *Racemosae* G.Don) described from the eastern Alps (Pustertal). According to Kalela^51^, the taxon differs from *C*. *norvegica* Retz. subsp. *norvegica* mainly in having longer utricles, narrower and shorter bracts, and wider leaves. Like in *C*. *buekii*, these are important taxonomic characters that proved variable and insufficient for supporting the validity of the taxon^52^, which has been also confirmed by molecular analyses^53^.

In our opinion, taxonomic studies on the genus *Carex*, based on both morphological and molecular analyses, should be carried out with reference to habitat conditions as well as biology and ecology of populations in the field; subsequently, the research can be complemented by herbarium data. Unfortunately, numerous taxonomist use only the latter and distinguish new species based on morphological and/or genetic variability of dried plants. Morphological examination of herbarium-held specimens of the section *Phaestoglochin* Dumort. have resulted in the description of 10 new taxa^54,55^. Due to the phenotypic plasticity observed, those descriptions raise controversies and are treated by some authors as synonyms of the already known species^56^. Molecular and morphological analyses within the section *Rhynchocystis* Dumort. revealed *C*. *agastachys* L.f., a neglected species from *C*. *pendula* agg.^57^. However, some of the morphological traits listed by Míguez et al.^57^ are hardly applicable to the identification of the species. Examination of the Czech specimens of *C*. *pendula* agg. showed the presence of individuals with a combination of vegetative traits of one species and the achene (nut) shape typical of another^58^.

Knowledge on the range of phenotypic plasticity in field populations seems to be of a key importance in taxonomic research. In the case of *C*. *buekii*, analysing herbarium-held specimens without considering the relationship between a trait and soil conditions could result in creating new entities which, on account of their morphological variability, would be difficult and/or impossible to identify. Taxonomic research should focus on an entity identifiable in the field (with a due consideration to its phenotypic plasticity), with specific ecological preferences, characterized by distinct biology and a specific distribution range.

## Conclusions

The central-European populations of *C*. *buekii* studied differed significantly in their morphology. Despite the differences in generative traits, important for the taxonomy of the genus *Carex* (mainly the size of the utricle and its beak), we do not think it appropriate to distinguish new taxa at the level of species, subspecies, or even variety. The morphological variability observed is most likely an effect of the species’ adaptation to the variable edaphic conditions on floodplains and at sites changed by anthropogenic activities, such as levees and channels. In this study, the soil potassium content was the only edaphic factor that significantly affected the morphological variability of *C*. *buekii*; nevertheless, interactions between potassium and other elements (e.g. nitrogen, magnesium, calcium) cannot be ruled out. Future studies should examine hydrological conditions and explore their effects on both *C*. *buekii* morphology and nutrient concentrations in the soil. In light of the results obtained, we posit that knowledge on the range of phenotypic plasticity in natural populations seems to be of a key importance in taxonomic studies.

## Supplementary Information

Supplementary Information.

## Data Availability

The datasets analysed during the current study available from the corresponding author on reasonable request.
