# Supplementary material for: Morphological variability of Carex buekii (Cyperaceae) as a function of soil conditions: a case study of the Central European populations
Source: Sci Rep. 2022 Jul 11;12:11761. doi: 10.1038/s41598-022-15894-0 (PMC9273646; doi:10.1038/s41598-022-15894-0)
Supplement: Supplementary file 1 — Supplementary Information. [file 41598_2022_15894_MOESM1_ESM.pdf]

Supplementary Table S1

Morphological variability of *Carex buekii* (Cyperaceae) as a function of soil conditions. A case study of the Central European populations

Helena Więcław[1], Beata Bosiacka[1], Richard Hrivnák[2], Zygmunt Dajdok[3], Attila Mesterházy[4], Jacob Koopman[5]

[1]University of Szczecin, Institute of Marine and Environmental Sciences, Adama Mickiewicza 18, PL-70-383, Szczecin, Poland; [2]Slovak Academy of Sciences, Institute of Botany, Plant Science and Biodiversity Center, Dúbravská cesta 9, SK-845 23 Bratislava, Slovakia; [3]University of Wrocław, Faculty of Biological Sciences, Department of Botany, Kanonia 6/8, PL-50-328, Wrocław, Poland; [4]Centre for Ecological Research, Wetland Ecology Research Group, Bem tér 18/C, H-4026 Debrecen, Hungary; [5]ul. Kochanowskiego 27, PL-73-200 Choszczno, Poland

e-mail: helena.wieclaw@usz.edu.pl

Information about the studied area (stands of *Carex buekii*)

Expalnation: org. mat. [%] – organic matter content; N [%] – nitrogen content; pH – soil pH; Ca [mg/kg] – calcium concentration; Mg [mg/kg] – magnesium concentration; P [mg/kg] – phosphorus concentration; K [mg/kg] – potassium concentration; ECe [dS/m] – electrolytic conductivity of the saturated soil extract.

Population no / Locality	Coordinates	Habitat type	Hydrological conditions	Soil properties	Elevation a. s. l.
			**Hungary**		
1 / Felsőcsatár	47°12'33"N, 16°25'55"E	banks of Pinka river in willow forest	banks of a river, distance from the open water 1-2 m. *C. buekii* phytocoenoses over the open water level: 1-2 m	org.mat=8.3; N=0.32; pH=5.9; Mg=177.3; P=127.3; K=73.9; Ca=2330.4.	245
2 / Körmend, Rába river	46°59'45"N, 16°35'56"E	banks of a small oxbow, edge of floodplain meadows	banks of an oxbow. In spring this oxbow is filled up by water and it dries out in June. distance from the open water 10 m. *C. buekii* phytocoenoses over the open water level: 2-3 m	org.mat=11.1; N=0.39; pH=5.6; Mg=223.5; P=110.7; K=118.0; Ca=2868.5.	185
3 / Kerkáskápolna, Kerka rivulet	46°46'52"N, 16°25'06"E	banks of an old riverbed in an alder forest	banks of a old stream, distance from the open water 1-2 m. *C. buekii* phytocoenoses over the open water level: 1-2 m	org.mat=8.6; N=0.28; pH=4.7; Mg=126.9; P=181.8; K=232.9; Ca=1270.8.	208
4 / Szentgyörgyvölgy	46°43'37"N, 16°23'57"E	on a hillside, near a spring, grazed mezophilous meadow near a spring	near spring, distance from the spring (some squermeters and the water is not open) 0-3 m. *C. buekii* phytocoenoses over the open water level: 0.3-0.5 m	org.mat=14.7; N=0.49; pH=5.5; Mg=116.8; P=149.1; K=93.1; Ca=1907.8.	194

5 / Bélapátfalva Bán stream	48°01'15"N, 20°19'44"E	former hay meadow, which was abandoned and covered with *Magnocaricion* stands	banks of a small stream, distance from the open water 10 m. *C. buekii* phytocoenoses over the open water level: 1-2 m	org.mat=11.2; N=0.44; pH=7.3; Mg=239.3; P=325.3; K=638.4; Ca=19296.8.	265
6 / Drégelypalánk	48°03'57"N, 19°02'48"E	banks of Ipoly river in willow forest near an alluvial hay meadow	banks of Ipoly river, distance from the open water 10 m. *C. buekii* phytocoenoses over the open water level: 2-3 m	org.mat=10.3; N=0.34; pH=6.4; Mg=219.8; P=327.0; K=220.0; Ca=3093.2.	126
**Poland**					
7 / Kotowice-Czernica	51°02'43.5"N, 17°13'18.1"E	floodplain of the Odra river	banks of the small watercourse flowing into the Odra River: distance from the open water - ca. 0-10 m; location of the *C. buekii* phytocoenoses over the open water level: ca. 0-4 m	org.mat=13.3; N=0.34; pH=5.1; Mg=54.3; P=898.6; K=142.9; Ca=901.8.	125
8 / Wrocław-Jarnołtów	51°07'29.9"N, 16°50'30.9"E	small depression on the Bystrzyca river floodplain	the floodplain terrace in the inter-embankment: distance from the nearest open water - ca. 60 m; location of the *C. buekii* phytocoenoses over the open water level: ca. 1-2 m	org.mat=13.5; N=0.42; pH=5.3; Mg=97.8; P=378.4; K=384.6; Ca=1522.9.	115
9 / Wrocław-Wojnów (Strachocin)	51°05'46.5"N, 17°08'07.9"E	small depression on the Odra river floodplain	the floodplain terrace in the inter-embankment: distance from the nearest open water - ca. 80 m; location of the *C. buekii* phytocoenoses over the open water level: ca. 2-3 m	org.mat=9.7; N=0.35; pH=4.6; Mg=69.7; P=266.0; K=372.6; Ca=931.2.	118
10 / Wrocław-Swojec	51°06'49.8"N, 17°06'40.4"E	banks of the navigation canal on the Odra river floodplain	vicinity of the shipping channel: distance from the open water - ca. 15 m; location of the *C. buekii* phytocoenoses over the open water level: ca. 2-3 m	org.mat=7.0; N=0.26; pH=5.1; Mg=39.2; P=201.0; K=493.8; Ca=1111.9.	113
11 / Stary Otok	50°58'41.7"N, 17°19'56.4"E	banks of the old river bed of Odra river	vicinity of the old river bed, outside the flood embankment: distance from the open water - ca. 10 m; location of the *C. buekii* phytocoenoses over the open water level: ca. 1-2 m	org.mat=3.9; N=0.13; pH=3.7; Mg=12.3; P=88.5; K=101.2; Ca=462.0.	126
12 / Wrocław-Świątniki	51°05'40.0"N, 17°06'31.1"E	floodplain of the Oława river	the floodplain terrace in the inter-embankment: distance from the nearest open water - ca. 30 m; location of the *C. buekii* phytocoenoses over the open water level: ca. 2-3 m	org.mat=14.8; N=0.48; pH=5.2; Mg=98.3; P=333.1; K=485.8; Ca=2500.6.	117
13 / Kruszyna	50°50'28.4"N, 17°32'42.7"E	floodbank in floodplain of the Odra river	internal slope of the flood embankment: distance from the open water - ca. 40 m; location of the *C. buekii* phytocoenoses over the open water level: ca. 4-5 m	org.mat=5.3; N=0.21; pH=4.3; Mg=48.2; P=120.3; K=290.7; Ca=471.8.	136

14 / Siechnice	51°02'17.1"N, 17°11'32.1"E	floodplain of the Odra river	the floodplain terrace in the inter-embankment: distance from the nearest open water - ca. 150 m; location of the *C. buekii* phytocoenoses over the open water level: ca. 2-2.5 m	org.mat=11.6; N=0.39; pH=4.7; Mg=103.1; P=110.3; K=265.0; Ca=1776.9.	121
15 / Warzyna	51°15'18.5"N, 16°46'44.6"E	floodplain of the Odra river	vicinity of the old river bed, outside the flood embankment: distance from the open water - ca. 20 m; location of the *C. buekii* phytocoenoses over the open water level: ca. 1-1.5 m	org.mat=5.7; N=0.20; pH=4.8; Mg=44.4; P=165.7; K=765.3; Ca=514.4.	106
16 / Kopanie	50°48'49.5"N, 17°38'09.7"E	floodplain of the Odra river	banks of the old river bed: distance from the open water - ca. 1 m; location of the *C. buekii* phytocoenoses over the open water level: ca. 0-1 m	org.mat=12.6; N=0.49; pH=5.7; Mg=91.0; P=782.6; K=285.1; Ca=2404.2.	138
**Slovakia**					
17 / Zvolen, Môťová	48°33'21.1"N, 19°11'28.9"E	bank of the Slatina river	banks of the river bed: distance from the open water - ca. 0.5–1 m; location of the *C. buekii* phytocoenoses over the open water level: ca. 0-2 m	org.mat=8.3; N=0.28; pH=4.6; Mg=74.3; P=274.7; K=360.5; Ca=1743.4.	307
18 / Slovenská Ľupča	48°45'15.1"N, 19°15'33.3"E	terrain depression on the Hron river floodplain	terrain depression: distance from the open water - ca. 25–30 m; location of the *C. buekii* phytocoenoses over the open water level: ca. 1-2 m	org.mat=8.7; N=0.30; pH=7.4; Mg=751.5; P=266.8; K=369.4; Ca=8536.0.	371
19 / Pstruša, near railway station	48°33'09.2"N, 19°18'55.5"E	terrain depression on the Slatina river floodplain	terrain depression: distance from the open water - ca. 120–150 m; location of the *C. buekii* phytocoenoses over the open water level: ca. 2-3 m	org.mat=8.3; N=0.31; pH=4.6; Mg=41.9; P=224.5; K=611.1; Ca=1137.5.	316
20 / Vyšný Skálnik	48°27'55.0"N, 19°57'36.4"E	terrain depression on the Rimava river floodplain	terrain depression: distance from the open water - ca. 170–180 m; location of the *C. buekii* phytocoenoses over the open water level: ca. 2-3 m	org.mat=14.4; N=0.44; pH=6.6; Mg=156.3; P=300.0; K=382.2; Ca=3298.6.	232
21 / Petrovce	48°11'37.5"N, 20°01'58.4"E	bank of the Mačací potok stream	banks of the stream bed: distance from the open water - ca. 0–3 m; location of the *C. buekii* phytocoenoses over the open water level: ca. 0-2 m	org.mat=5.5; N=0.21; pH=7.6; Mg=493.0; P=454.7; K=676.9; Ca=9106.9.	225
22 / Tomášovce	48°24'52.8"N, 19°35'31.3"E	bank of cannalized stream	banks of the stream bed: distance from the open water - ca. 0–2 m; location of the *C. buekii* phytocoenoses over the open water level: ca. 0-1m	org.mat=8.3; N=0.28; pH=6.2; Mg=145.8; P=269.0; K=696.2; Ca=1807.3.	220
23 / Moškovec	48°56'23.4"N, 18°49'47.5"E	bank of the Turiec river	banks of the river bed: distance from the open water - ca. 0–7 m; location of the *C. buekii*	org.mat=12.9; N=0.46; pH=7.3; Mg=2056.0;	445

			phytocoenoses over the open water level: ca. 0-2 m	P=379.3; K=220.0; Ca=20943.1.	
24 / Breznička, Červeň settlement	48°24'42.6"N, 19°44'10.7"E	bank of the Ipeľ river	banks of the river bed: distance from the open water - ca. 0–5 m; location of the *C. buekii* phytocoenoses over the open water level: ca. 0-4 m	org.mat=2.6; N=0.1; pH=6.0; Mg=38.8; P=323.1; K=119.6; Ca=731.0.	210
25 / Veľká Lúka	48°37'32.4"N, 19°09'33.5"E	bank of the canal near road,	banks of the canal bed: distance from the open water - ca. 0–3 m; location of the *C. buekii* phytocoenoses over the open water level: ca. 0-2 m	org.mat=11.6; N=0.40; pH=7.0; Mg=296.0; P=307.8; K=534.8; Ca=6679.9.	304
26 / Šávoľ	48°18'04.9"N, 19°48'50.6"E	bank of the canalized Suchá stream	banks of the stream bed: distance from the open water - ca. 0–5 m; location of the *C. buekii* phytocoenoses over the open water level: ca. 0-4 m	org.mat=8.1; N=0.27; pH=7.3; Mg=240.0; P=392.8; K=545.2; Ca=4472.6.	188

Supplementary Table S2

Morphological variability of *Carex buekii* (Cyperaceae) as a function of soil conditions. A case study of the Central European populations

Helena Więcław[1], Beata Bosiacka[1], Richard Hrivnák[2], Zygmunt Dajdok[3], Attila Mesterházy[4], Jacob Koopman[5]

[1]University of Szczecin, Institute of Marine and Environmental Sciences, Adama Mickiewicza 18, PL-70-383, Szczecin, Poland; [2]Slovak Academy of Sciences, Institute of Botany, Plant Science and Biodiversity Center, Dúbravská cesta 9, SK-845 23 Bratislava, Slovakia; [3]University of Wrocław, Faculty of Biological Sciences, Department of Botany, Kanonia 6/8, PL-50-328, Wrocław, Poland; [4]Centre for Ecological Research, Wetland Ecology Research Group, Bem tér 18/C, H-4026 Debrecen, Hungary: [5]ul. Kochanowskiego 27, PL-73-200 Choszczno, Poland

e-mail: helena.wieclaw@usz.edu.pl

Results of Spearman's rank association test between morphological traits of *Carex buekii* and soil properties.
Significant differences ($p \leq 0.05$) have been marked with bold.

Explanations: CH – Culm height; CLL – Cauline leaf length; CLW – Cauline leaf width; NFS – Number of female spikes; NMS – Number of male spikes; IL – Inflorescence length; MSL – Male spike length; MSW – Male spike width; FSL – Female spike length; FSW – Female spike width; PL – Peduncle length; BL – Bract length; UL – Utricle length; UBL – Utricle beak length; UBL/UL – Ratio of beak length to utricle length; GL – Glume length; org.mat. – organic matter; N –nitrogen; pH – soil pH; P – phosphorus; K – potassium; Mg – magnesium; Ca – calcium.

Soil properties	Morphological traits															
	**CH**	**CLL**	**CLW**	**NFS**	**NMS**	**IL**	**MSL**	**MSW**	**FSL**	**FSW**	**PL**	**BL**	**UL**	**UBL**	**UBL/UL**	**GL**
org.mat.	-0.143	-0.052	-0.124	0.171	-0.105	0.027	-0.033	0.221	0.036	0.317	-0.315	-0.023	0.199	-0.047	-0.252	-0.211
N	-0.202	-0.129	-0.149	0.099	-0.054	0.001	-0.008	0.183	0.028	0.261	-0.315	-0.091	0.139	-0.142	-0.271	-0.234
pH	0.082	0.191	**0.433**	-0.347	-0.048	0.221	**0.465**	-0.142	0.328	0.003	-0.304	0.191	-0.141	-0.075	0.103	0.173
Mg	-0.101	0.015	0.177	**-0.401**	0.064	0.093	0.201	-0.119	-0.032	0.039	**-0.394**	0.108	-0.127	-0.237	-0.005	0.004
P	0.202	0.312	**0.432**	0.059	-0.021	0.387	0.202	-0.021	0.233	0.279	-0.111	**0.448**	0.146	0.262	0.062	0.177
K	**0.635**	**0.447**	**0.643**	-0.035	0.319	0.243	0.218	0.044	0.186	0.015	-0.142	**0.448**	0.094	0.104	-0.047	0.316
Ca	-0.014	0.092	0.284	-0.317	0.002	0.142	0.263	-0.107	0.076	0.067	**-0.418**	0.151	-0.013	-0.168	-0.045	0.019

Supplementary Table S3

Morphological variability of *Carex buekii* (Cyperaceae) as a function of soil conditions. A case study of the Central European populations

Helena Więcław[1], Beata Bosiacka[1], Richard Hrivnák[2], Zygmunt Dajdok[3], Attila Mesterházy[4], Jacob Koopman[5]
[1]University of Szczecin, Institute of Marine and Environmental Sciences, Adama Mickiewicza 18, PL-70-383, Szczecin, Poland; [2]Slovak Academy of Sciences, Institute of Botany, Plant Science and Biodiversity Center, Dúbravská cesta 9, SK-845 23 Bratislava, Slovakia; [3]University of Wrocław, Faculty of Biological Sciences, Department of Botany, Kanonia 6/8, PL-50-328, Wrocław, Poland; [4]Centre for Ecological Research, Wetland Ecology Research Group, Bem tér 18/C, H-4026 Debrecen, Hungary; [5]ul. Kochanowskiego 27, PL-73-200 Choszczno, Poland

Results of Kruskal-Wallis test and post-hoc Dunn's multiple comparisons test, showing significance of differences in morphological traits of *Carex buekii* populations.
Explanations: *p* – significance level; significant differences ($p \leq 0.05$) have been marked with bold. 1, 2,3, …26 – population numer; CH – Culm height; CLL – Cauline leaf length; CLW – Cauline leaf width; NFS – Number of female spikes; NMS – Number of male spikes; IL – Inflorescence length; MSL – Male spike length; MSW – Male spike width; FSL – Female spike length; FSW – Female spike width; PL – Peduncle length; BL – Bract length; UL – Utricle length; UBL – Utricle beak length; UBL/UL – Ratio of beak length to utricle length; GL – Glume length.

Traits	Kruskal–Wallis test		Dunn's multiple comparisons test																								
			1-2	1-3	1-4	1-5	1-6	1-7	1-8	1-9	1-10	1-11	1-12	1-13	1-14	1-15	1-16	1-17	1-18	1-19	1-20	1-21	1-22	1-23	1-24	1-25	1-26
	H	*p*	*p*	*p*	*p*	*p*	*p*	*p*	*p*	*p*	*p*	*p*	*p*	*p*	*p*	*p*	*p*	*p*	*p*	*p*	*p*	*p*	*p*	*p*	*p*	*p*	*p*
CH	309.9	**0.00**	1.00	1.00	0.50	1.00	1.00	1.00	1.00	0.41	**0.00**	1.00	**0.01**	1.00	**0.04**	**0.00**	1.00	0.63	1.00	1.00	1.00	0.57	**0.02**	1.00	1.00	**0.01**	0.17
CLL	259.9	**0.00**	**0.00**	**0.00**	**0.05**	0.86	1.00	1.00	1.00	1.00	1.00	**0.02**	1.00	0.26	1.00	1.00	1.00	1.00	1.00	1.00	1.00	1.00	1.00	**0.00**	1.00	1.00	1.00
CLW	255.3	**0.00**	1.00	1.00	1.00	1.00	1.00	1.00	1.00	1.00	1.00	1.00	1.00	1.00	1.00	1.00	1.00	0.07	**0.00**	0.33	**0.00**	**0.00**	**0.00**	1.00	**0.01**	0.44	**0.00**
NFS	152.4	**0.00**	1.00	1.00	1.00	1.00	1.00	**0.05**	1.00	**0.05**	1.00	1.00	**0.00**	1.00	1.00	1.00	1.00	1.00	1.00	1.00	1.00	1.00	0.06	1.00	1.00	1.00	1.00
NMS	141.8	**0.00**	1.00	**0.01**	**0.02**	**0.00**	**0.00**	1.00	**0.02**	**0.01**	0.12	1.00	1.00	**0.01**	1.00	1.00	1.00	**0.00**	1.00	**0.01**	0.07	**0.00**	**0.00**	**0.04**	0.14	**0.00**	1.00
IL	175.6	**0.00**	0.36	**0.00**	0.06	0.38	1.00	1.00	1.00	1.00	1.00	**0.02**	1.00	1.00	0.15	1.00	1.00	1.00	1.00	0.09	1.00	1.00	1.00	1.00	1.00	1.00	1.00
MSL	175.8	**0.00**	**0.01**	**0.00**	**0.00**	**0.00**	1.00	**0.01**	0.81	0.96	1.00	**0.00**	1.00	**0.00**	**0.04**	1.00	0.20	**0.00**	0.59	**0.00**	0.14	**0.03**	1.00	**0.05**	0.87	1.00	**0.00**
MSW	182.4	**0.00**	0.14	1.00	1.00	**0.01**	1.00	**0.00**	1.00	1.00	1.00	1.00	1.00	1.00	0.54	1.00	**0.02**	1.00	1.00	0.37	1.00	1.00	**0.00**	1.00	1.00	1.00	1.00
FSL	261.9	**0.00**	**0.00**	**0.00**	**0.00**	1.00	**0.00**	0.55	1.00	**0.00**	1.00	**0.00**	1.00	**0.01**	0.23	1.00	1.00	**0.00**	**0.00**	**0.00**	1.00	1.00	1.00	**0.00**	1.00	0.10	**0.00**
FSW	216.3	**0.00**	1.00	**0.00**	1.00	1.00	**0.00**	1.00	**0.00**	1.00	1.00	1.00	0.09	1.00	**0.00**	**0.05**	**0.00**	**0.00**	**0.00**	**0.02**	1.00	1.00	**0.00**	**0.00**	0.45	**0.00**	1.00
PL	150.2	**0.00**	1.00	1.00	1.00	**0.00**	**0.00**	1.00	1.00	1.00	1.00	1.00	1.00	1.00	**0.00**	1.00	1.00	1.00	1.00	1.00	**0.00**	1.00	1.00	0.18	1.00	1.00	1.00
BL	170.0	**0.00**	1.00	1.00	1.00	1.00	1.00	1.00	1.00	**0.03**	1.00	1.00	0.12	1.00	1.00	1.00	1.00	1.00	0.06	1.00	1.00	0.97	1.00	1.00	1.00	1.00	1.00
UL	338.6	**0.00**	1.00	**0.00**	1.00	**0.00**	**0.00**	1.00	**0.00**	1.00	0.13	**0.00**	**0.00**	1.00	**0.00**	**0.00**	**0.00**	**0.00**	**0.00**	**0.00**	**0.00**	1.00	0.56	**0.00**	**0.00**	**0.00**	1.00
UBL	325.6	**0.00**	1.00	**0.00**	1.00	1.00	0.51	1.00	**0.00**	1.00	1.00	**0.00**	**0.00**	1.00	1.00	**0.00**	0.11	**0.00**	1.00	0.10	**0.01**	0.18	**0.00**	**0.00**	**0.00**	**0.00**	1.00
UBL/UL	309.9	**0.00**	1.00	1.00	0.33	**0.00**	1.00	1.00	1.00	1.00	1.00	1.00	1.00	1.00	1.00	1.00	1.00	1.00	**0.00**	1.00	1.00	1.00	1.00	**0.00**	0.18	1.00	1.00
GL	332.8	**0.00**	1.00	1.00	1.00	**0.00**	**0.00**	1.00	**0.00**	0.06	0.34	**0.00**	**0.00**	**0.00**	**0.01**	**0.00**	1.00	**0.00**	**0.00**	1.00	**0.02**	**0.00**	**0.00**	**0.00**	**0.00**	1.00	0.97

Traits	Dunn's multiple comparisons test																										
	2-3	2-4	2-5	2-6	2-7	2-8	2-9	2-10	2-11	2-12	2-13	2-14	2-15	2-16	2-17	2-18	2-19	2-20	2-21	2-22	2-23	2-24	2-25	2-26	3-4	3-5	3-6
	p	*p*	*p*	*p*	*p*	*p*	*p*	*p*	*p*	*p*	*p*	*p*	*p*	*p*	*p*	*p*	*p*	*p*	*p*	*p*	*p*	*p*	*p*	*p*	*p*	*p*	*p*
CH	1.00	1.00	0.46	**0.00**	**0.01**	1.00	**0.00**	**0.00**	1.00	**0.00**	1.00	**0.00**	**0.00**	1.00	**0.00**	**0.00**	0.05	**0.00**	**0.00**	**0.00**	1.00	**0.00**	**0.00**	**0.00**	1.00	0.35	**0.00**
CLL	1.00	1.00	1.00	1.00	**0.00**	1.00	**0.00**	**0.00**	1.00	**0.00**	1.00	**0.00**	**0.01**	1.00	0.12	**0.00**	**0.00**	**0.00**	**0.00**	**0.00**	1.00	0.06	1.00	**0.00**	1.00	1.00	1.00
CLW	1.00	1.00	**0.00**	1.00	0.17	0.26	0.09	1.00	1.00	**0.00**	1.00	0.30	**0.00**	0.74	**0.00**	**0.00**	**0.00**	**0.00**	**0.00**	**0.00**	**0.01**	**0.00**	**0.00**	**0.00**	1.00	**0.00**	1.00
NFS	1.00	1.00	1.00	1.00	0.07	1.00	0.07	1.00	1.00	**0.00**	1.00	1.00	1.00	1.00	1.00	1.00	1.00	1.00	1.00	0.09	1.00	1.00	1.00	1.00	1.00	1.00	1.00
NMS	1.00	1.00	**0.03**	1.00	1.00	1.00	1.00	1.00	1.00	1.00	1.00	1.00	1.00	1.00	1.00	1.00	1.00	1.00	1.00	1.00	1.00	1.00	1.00	1.00	1.00	1.00	1.00
IL	1.00	1.00	1.00	1.00	0.82	1.00	**0.00**	0.12	1.00	**0.01**	1.00	1.00	0.16	1.00	1.00	**0.01**	1.00	1.00	1.00	1.00	**0.00**	1.00	0.64	1.00	1.00	1.00	**0.01**
MSL	0.14	1.00	1.00	1.00	1.00	1.00	1.00	**0.05**	1.00	1.00	1.00	1.00	1.00	1.00	1.00	1.00	0.90	1.00	1.00	1.00	1.00	1.00	0.31	1.00	1.00	0.25	**0.00**
MSW	1.00	1.00	1.00	1.00	1.00	1.00	**0.01**	1.00	1.00	0.86	1.00	1.00	1.00	1.00	0.17	**0.01**	1.00	1.00	**0.00**	1.00	**0.02**	0.24	1.00	1.00	1.00	1.00	1.00
FSL	1.00	1.00	1.00	1.00	1.00	1.00	1.00	**0.00**	1.00	1.00	1.00	1.00	**0.03**	1.00	1.00	1.00	1.00	1.00	0.20	**0.05**	1.00	**0.00**	1.00	1.00	1.00	**0.00**	0.35
FSW	**0.00**	1.00	1.00	**0.00**	1.00	**0.00**	1.00	1.00	1.00	0.06	1.00	**0.01**	**0.03**	**0.00**	**0.00**	**0.00**	**0.02**	1.00	1.00	**0.00**	**0.00**	0.32	**0.00**	1.00	1.00	0.15	1.00
PL	1.00	1.00	**0.00**	**0.00**	1.00	1.00	1.00	1.00	1.00	1.00	1.00	**0.00**	1.00	1.00	1.00	1.00	1.00	**0.00**	1.00	1.00	0.08	1.00	1.00	1.00	1.00	**0.02**	**0.00**
BL	1.00	1.00	1.00	**0.00**	0.06	1.00	**0.00**	**0.02**	1.00	**0.00**	0.07	1.00	**0.00**	0.47	**0.01**	**0.00**	1.00	**0.00**	**0.00**	1.00	**0.00**	1.00	**0.00**	**0.00**	1.00	1.00	**0.00**
UL	**0.00**	1.00	**0.00**	**0.00**	0.38	**0.00**	1.00	**0.04**	**0.00**	**0.00**	0.38	**0.00**	**0.00**	**0.00**	**0.00**	**0.00**	**0.00**	**0.00**	1.00	0.15	**0.00**	**0.00**	**0.00**	1.00	**0.02**	1.00	1.00
UBL	**0.00**	1.00	1.00	0.19	0.98	**0.00**	1.00	1.00	**0.00**	**0.00**	1.00	1.00	**0.00**	**0.04**	**0.00**	1.00	**0.04**	**0.00**	0.06	**0.00**	**0.00**	**0.00**	**0.00**	1.00	**0.00**	**0.00**	1.00
UBL/UL	1.00	0.34	**0.00**	1.00	1.00	1.00	1.00	1.00	1.00	1.00	1.00	1.00	1.00	1.00	1.00	**0.00**	1.00	1.00	1.00	1.00	**0.00**	0.25	1.00	1.00	0.99	**0.00**	1.00
GL	1.00	1.00	**0.00**	**0.00**	1.00	**0.00**	0.06	0.32	**0.00**	**0.00**	**0.01**	**0.01**	**0.00**	1.00	**0.00**	**0.00**	1.00	**0.02**	**0.00**	**0.00**	**0.00**	**0.00**	1.00	0.90	1.00	0.75	**0.00**

Traits	Dunn's multiple comparisons test																										
	3-7	3-8	3-9	3-10	3-11	3-12	3-13	3-14	3-15	3-16	3-17	3-18	3-19	3-20	3-21	3-22	3-23	3-24	3-25	3-26	4-5	4-6	4-7	4-8	4-9	4-10	4-11
	p	*p*	*p*	*p*	*p*	*p*	*p*	*p*	*p*	*p*	*p*	*p*	*p*	*p*	*p*	*p*	*p*	*p*	*p*	*p*	*p*	*p*	*p*	*p*	*p*	*p*	*p*
CH	**0.01**	1.00	**0.00**	**0.00**	1.00	**0.00**	1.00	**0.00**	**0.00**	1.00	**0.00**	**0.02**	**0.03**	**0.00**	**0.00**	**0.00**	1.00	**0.00**	**0.00**	**0.00**	0.06	**0.00**	**0.00**	1.00	**0.00**	**0.00**	1.00
CLL	**0.00**	1.00	**0.00**	**0.00**	1.00	**0.00**	1.00	**0.02**	0.06	1.00	1.00	**0.00**	**0.00**	**0.00**	**0.00**	0.06	1.00	0.53	1.00	**0.00**	1.00	1.00	**0.01**	1.00	**0.00**	**0.05**	1.00
CLW	0.15	0.23	0.07	1.00	1.00	**0.00**	1.00	0.26	**0.00**	0.65	**0.00**	**0.00**	**0.00**	**0.00**	**0.00**	**0.00**	**0.01**	**0.00**	**0.00**	**0.00**	1.00	1.00	1.00	1.00	1.00	1.00	1.00
NFS	0.07	1.00	0.09	1.00	1.00	**0.00**	1.00	1.00	1.00	1.00	1.00	1.00	1.00	1.00	1.00	0.09	1.00	1.00	1.00	1.00	1.00	1.00	**0.02**	1.00	**0.02**	1.00	1.00
NMS	0.21	1.00	1.00	1.00	1.00	**0.03**	1.00	1.00	1.00	1.00	1.00	1.00	1.00	1.00	1.00	1.00	1.00	1.00	1.00	1.00	1.00	1.00	0.35	1.00	1.00	1.00	1.00
IL	**0.00**	1.00	**0.00**	**0.00**	1.00	**0.00**	0.08	1.00	**0.00**	0.07	**0.00**	**0.00**	1.00	**0.01**	**0.00**	0.38	**0.00**	**0.00**	**0.00**	**0.00**	1.00	1.00	0.16	1.00	**0.00**	**0.02**	1.00
MSL	1.00	**0.04**	**0.01**	**0.00**	1.00	**0.00**	1.00	1.00	**0.00**	0.10	**0.02**	**0.00**	1.00	**0.00**	**0.00**	**0.00**	**0.00**	**0.00**	**0.00**	0.08	1.00	1.00	1.00	1.00	1.00	**0.00**	1.00
MSW	1.00	1.00	0.14	1.00	1.00	1.00	1.00	1.00	1.00	1.00	1.00	0.12	1.00	1.00	**0.03**	0.08	1.00	1.00	1.00	0.08	0.64	1.00	**0.04**	1.00	1.00	1.00	1.00
FSL	0.07	**0.00**	1.00	**0.00**	1.00	**0.00**	1.00	1.00	**0.00**	**0.00**	0.70	1.00	1.00	**0.00**	**0.00**	**0.00**	**0.04**	**0.00**	**0.00**	1.00	1.00	1.00	1.00	1.00	1.00	**0.00**	1.00
FSW	1.00	1.00	1.00	0.36	1.00	1.00	0.96	1.00	1.00	1.00	1.00	1.00	1.00	1.00	**0.02**	1.00	1.00	1.00	1.00	1.00	1.00	1.00	1.00	0.80	1.00	1.00	1.00
PL	1.00	1.00	1.00	1.00	1.00	1.00	1.00	**0.01**	1.00	1.00	1.00	1.00	1.00	**0.03**	1.00	1.00	1.00	1.00	1.00	1.00	1.00	0.15	1.00	1.00	1.00	1.00	1.00
BL	**0.05**	1.00	**0.00**	**0.02**	1.00	**0.00**	0.06	1.00	**0.00**	0.44	**0.00**	**0.00**	1.00	**0.00**	**0.00**	1.00	**0.00**	1.00	**0.00**	**0.00**	1.00	**0.01**	0.17	1.00	**0.00**	0.07	1.00
UL	1.00	1.00	1.00	1.00	1.00	1.00	1.00	1.00	1.00	1.00	1.00	1.00	1.00	1.00	**0.01**	1.00	0.10	1.00	1.00	**0.00**	**0.02**	**0.01**	1.00	**0.00**	1.00	1.00	**0.01**
UBL	1.00	1.00	1.00	1.00	1.00	1.00	0.72	1.00	1.00	1.00	1.00	1.00	1.00	1.00	1.00	1.00	**0.02**	1.00	1.00	**0.00**	1.00	0.19	0.93	**0.00**	1.00	1.00	**0.00**
UBL/UL	1.00	1.00	1.00	1.00	1.00	1.00	1.00	1.00	1.00	1.00	1.00	**0.01**	1.00	1.00	1.00	1.00	**0.00**	**0.04**	1.00	1.00	1.00	1.00	1.00	1.00	1.00	1.00	**0.00**
GL	1.00	**0.00**	1.00	1.00	**0.03**	**0.00**	1.00	1.00	**0.00**	1.00	0.35	0.12	1.00	1.00	**0.00**	**0.00**	**0.00**	**0.00**	1.00	1.00	1.00	**0.00**	1.00	0.24	1.00	1.00	1.00

Traits	Dunn's multiple comparisons test																										
	4-12	4-13	4-14	4-15	4-16	4-17	4-18	4-19	4-20	4-21	4-22	4-23	4-24	4-25	4-26	5-6	5-7	5-8	5-9	5-10	5-11	5-12	5-13	5-14	5-15	5-16	5-17
	p	*p*	*p*	*p*	*p*	*p*	*p*	*p*	*p*	*p*	*p*	*p*	*p*	*p*	*p*	*p*	*p*	*p*	*p*	*p*	*p*	*p*	*p*	*p*	*p*	*p*	*p*
CH	**0.00**	1.00	**0.00**	**0.00**	1.00	**0.00**	**0.00**	**0.00**	**0.00**	**0.00**	**0.00**	1.00	**0.00**	**0.00**	**0.00**	1.00	1.00	1.00	1.00	**0.03**	1.00	0.08	1.00	0.28	**0.00**	1.00	1.00
CLL	**0.00**	1.00	0.28	0.92	1.00	1.00	**0.00**	0.12	**0.00**	**0.00**	1.00	1.00	1.00	1.00	**0.00**	1.00	0.26	1.00	**0.05**	0.75	1.00	**0.00**	1.00	1.00	1.00	1.00	1.00
CLW	1.00	1.00	1.00	1.00	1.00	**0.01**	**0.00**	**0.03**	**0.00**	**0.00**	**0.00**	1.00	**0.00**	0.06	**0.00**	1.00	1.00	1.00	1.00	1.00	1.00	1.00	1.00	1.00	1.00	1.00	1.00
NFS	**0.00**	1.00	1.00	1.00	1.00	1.00	1.00	1.00	0.50	1.00	**0.02**	1.00	1.00	1.00	1.00	1.00	**0.00**	1.00	**0.01**	1.00	1.00	**0.00**	1.00	0.58	1.00	0.49	1.00
NMS	0.06	1.00	1.00	1.00	1.00	1.00	1.00	1.00	1.00	1.00	1.00	1.00	1.00	1.00	1.00	1.00	**0.00**	1.00	1.00	1.00	**0.01**	**0.00**	1.00	0.84	0.06	**0.01**	1.00
IL	**0.00**	1.00	1.00	**0.02**	1.00	0.50	**0.00**	1.00	1.00	0.26	1.00	**0.00**	1.00	0.09	0.33	1.00	0.86	1.00	**0.00**	0.13	1.00	**0.01**	1.00	1.00	0.16	1.00	1.00
MSL	0.29	1.00	1.00	1.00	1.00	1.00	1.00	1.00	1.00	1.00	0.34	1.00	1.00	**0.01**	1.00	1.00	1.00	1.00	1.00	**0.02**	1.00	1.00	1.00	1.00	1.00	1.00	1.00
MSW	1.00	1.00	1.00	1.00	0.74	1.00	1.00	1.00	1.00	0.88	0.22	1.00	1.00	1.00	1.00	1.00	1.00	1.00	**0.00**	0.70	1.00	0.10	0.49	1.00	1.00	1.00	**0.01**
FSL	1.00	1.00	1.00	**0.02**	1.00	1.00	1.00	1.00	**0.00**	0.16	**0.04**	1.00	**0.00**	1.00	1.00	1.00	1.00	1.00	**0.00**	1.00	1.00	1.00	1.00	1.00	1.00	1.00	1.00
FSW	1.00	1.00	1.00	1.00	**0.01**	1.00	1.00	1.00	1.00	1.00	1.00	**0.01**	1.00	1.00	1.00	**0.01**	1.00	**0.01**	1.00	1.00	1.00	1.00	1.00	0.95	1.00	**0.00**	**0.00**
PL	1.00	1.00	0.63	1.00	1.00	1.00	1.00	1.00	1.00	1.00	1.00	1.00	1.00	1.00	1.00	1.00	1.00	**0.00**	1.00	**0.00**	**0.00**	1.00	1.00	1.00	**0.01**	0.06	**0.00**
BL	**0.00**	0.20	1.00	**0.01**	1.00	**0.02**	**0.00**	1.00	**0.01**	**0.00**	1.00	**0.02**	1.00	**0.00**	**0.00**	0.62	1.00	1.00	**0.00**	1.00	1.00	**0.01**	1.00	1.00	0.51	1.00	1.00
UL	**0.00**	1.00	0.06	**0.00**	**0.01**	**0.00**	**0.00**	**0.00**	0.30	1.00	1.00	**0.00**	0.17	1.00	1.00	1.00	1.00	1.00	1.00	1.00	1.00	1.00	1.00	1.00	1.00	1.00	1.00
UBL	**0.00**	1.00	1.00	**0.00**	**0.04**	**0.00**	1.00	**0.03**	**0.00**	0.06	**0.00**	**0.00**	**0.00**	**0.00**	1.00	**0.00**	**0.04**	**0.00**	0.88	0.13	**0.00**	**0.00**	1.00	1.00	**0.00**	**0.00**	**0.00**
UBL/UL	1.00	1.00	1.00	**0.00**	1.00	0.53	1.00	1.00	0.17	**0.00**	**0.00**	**0.00**	**0.00**	**0.00**	1.00	0.38	**0.00**	1.00	**0.04**	0.09	**0.00**	**0.00**	1.00	1.00	**0.00**	0.14	**0.00**
GL	0.20	1.00	1.00	**0.00**	1.00	1.00	1.00	1.00	1.00	**0.00**	**0.00**	**0.00**	**0.04**	1.00	1.00	1.00	**0.02**	1.00	1.00	1.00	1.00	1.00	1.00	1.00	1.00	1.00	1.00

Traits	Dunn's multiple comparisons test																										
	5-18	5-19	5-20	5-21	5-22	5-23	5-24	5-25	5-26	6-7	6-8	6-9	6-10	6-11	6-12	6-13	6-14	6-15	6-16	6-17	6-18	6-19	6-20	6-21	6-22	6-23	6-24
	p	*p*	*p*	*p*	*p*	*p*	*p*	*p*	*p*	*p*	*p*	*p*	*p*	*p*	*p*	*p*	*p*	*p*	*p*	*p*	*p*	*p*	*p*	*p*	*p*	*p*	*p*
CH	1.00	1.00	1.00	1.00	0.21	1.00	1.00	**0.01**	1.00	1.00	1.00	1.00	0.23	0.32	0.61	1.00	1.00	**0.01**	1.00	1.00	1.00	1.00	1.00	1.00	1.00	1.00	1.00
CLL	**0.03**	1.00	0.06	**0.00**	1.00	1.00	1.00	1.00	**0.00**	0.62	1.00	0.11	1.00	1.00	**0.00**	1.00	1.00	1.00	1.00	1.00	0.07	1.00	0.13	**0.00**	1.00	1.00	1.00
CLW	1.00	1.00	1.00	**0.04**	1.00	1.00	1.00	1.00	1.00	1.00	1.00	1.00	1.00	1.00	1.00	1.00	1.00	1.00	1.00	**0.03**	**0.00**	0.17	**0.00**	**0.00**	**0.00**	1.00	**0.00**
NFS	1.00	0.73	0.14	1.00	**0.00**	1.00	1.00	1.00	1.00	0.99	1.00	0.98	1.00	1.00	**0.03**	1.00	1.00	1.00	1.00	1.00	1.00	1.00	1.00	1.00	1.00	1.00	1.00
NMS	**0.01**	0.97	0.48	1.00	1.00	0.40	0.78	1.00	**0.00**	0.08	1.00	1.00	1.00	1.00	**0.01**	1.00	1.00	1.00	1.00	1.00	1.00	1.00	1.00	1.00	1.00	1.00	1.00
IL	**0.01**	1.00	1.00	1.00	1.00	**0.00**	1.00	0.66	1.00	1.00	1.00	0.48	1.00	1.00	0.97	1.00	1.00	1.00	1.00	1.00	1.00	1.00	1.00	1.00	1.00	1.00	1.00
MSL	1.00	1.00	1.00	1.00	1.00	1.00	1.00	0.10	1.00	1.00	1.00	1.00	1.00	1.00	1.00	1.00	1.00	1.00	1.00	1.00	1.00	**0.00**	1.00	1.00	1.00	1.00	1.00
MSW	**0.00**	1.00	0.55	**0.00**	1.00	**0.00**	**0.01**	1.00	1.00	0.07	1.00	1.00	1.00	1.00	1.00	1.00	1.00	1.00	1.00	1.00	1.00	1.00	1.00	**0.01**	0.37	1.00	1.00
FSL	1.00	**0.00**	1.00	1.00	1.00	1.00	1.00	1.00	0.09	1.00	1.00	0.16	**0.05**	1.00	1.00	1.00	1.00	0.59	1.00	1.00	1.00	0.87	**0.00**	1.00	1.00	1.00	**0.01**
FSW	**0.01**	1.00	1.00	1.00	**0.01**	**0.00**	1.00	0.15	1.00	1.00	1.00	0.66	**0.04**	1.00	1.00	0.13	1.00	1.00	1.00	1.00	1.00	1.00	0.23	**0.00**	1.00	1.00	1.00
PL	1.00	**0.04**	1.00	1.00	0.51	1.00	**0.01**	1.00	**0.00**	0.13	**0.00**	1.00	**0.00**	**0.00**	1.00	0.52	1.00	**0.00**	**0.00**	**0.00**	0.12	**0.00**	1.00	0.09	**0.02**	1.00	**0.00**
BL	**0.00**	1.00	1.00	0.06	1.00	1.00	1.00	0.45	0.34	1.00	1.00	1.00	1.00	0.36	1.00	1.00	1.00	1.00	1.00	1.00	1.00	0.29	1.00	1.00	0.51	1.00	1.00
UL	1.00	1.00	1.00	**0.01**	1.00	0.07	1.00	1.00	**0.00**	1.00	0.71	1.00	1.00	1.00	1.00	1.00	1.00	1.00	1.00	1.00	1.00	1.00	1.00	**0.00**	0.84	**0.01**	1.00
UBL	0.13	**0.00**	**0.00**	**0.00**	**0.00**	**0.00**	**0.00**	**0.00**	1.00	1.00	1.00	1.00	1.00	0.34	0.20	1.00	1.00	0.34	1.00	1.00	1.00	1.00	1.00	1.00	1.00	**0.00**	**0.01**
UBL/UL	1.00	**0.02**	**0.00**	**0.00**	**0.00**	**0.00**	**0.00**	**0.00**	1.00	1.00	1.00	1.00	1.00	0.07	1.00	1.00	1.00	**0.03**	1.00	1.00	1.00	1.00	1.00	**0.01**	**0.01**	**0.00**	**0.00**
GL	1.00	0.61	1.00	1.00	0.27	0.21	0.06	0.14	1.00	**0.00**	1.00	1.00	0.59	1.00	1.00	1.00	1.00	1.00	**0.01**	0.99	1.00	**0.00**	0.06	1.00	1.00	1.00	1.00

Traits	Dunn's multiple comparisons test																										
	6-25	6-26	7-8	7-9	7-10	7-11	7-12	7-13	7-14	7-15	7-16	7-17	7-18	7-19	7-20	7-21	7-22	7-23	7-24	7-25	7-26	8-9	8-10	8-11	8-12	8-13	8-14
	p	*p*	*p*	*p*	*p*	*p*	*p*	*p*	*p*	*p*	*p*	*p*	*p*	*p*	*p*	*p*	*p*	*p*	*p*	*p*	*p*	*p*	*p*	*p*	*p*	*p*	*p*
CH	0.12	1.00	1.00	1.00	1.00	0.37	1.00	1.00	1.00	0.24	1.00	1.00	1.00	1.00	1.00	1.00	1.00	1.00	1.00	1.00	1.00	1.00	**0.03**	1.00	0.07	1.00	0.21
CLL	1.00	**0.00**	0.43	1.00	1.00	**0.01**	1.00	0.08	1.00	1.00	0.51	1.00	1.00	1.00	1.00	1.00	1.00	**0.00**	1.00	0.59	1.00	0.09	1.00	1.00	**0.00**	1.00	1.00
CLW	0.25	**0.00**	1.00	1.00	1.00	1.00	1.00	1.00	1.00	1.00	1.00	1.00	1.00	1.00	0.40	**0.01**	0.56	1.00	1.00	1.00	1.00	1.00	1.00	1.00	1.00	1.00	1.00
NFS	1.00	1.00	0.34	1.00	1.00	1.00	1.00	1.00	1.00	0.86	1.00	0.62	1.00	1.00	1.00	**0.00**	1.00	1.00	1.00	**0.00**	0.83	0.33	1.00	1.00	**0.01**	1.00	1.00
NMS	1.00	1.00	0.28	0.21	1.00	1.00	1.00	0.17	1.00	1.00	1.00	**0.00**	1.00	0.51	1.00	0.07	0.06	1.00	1.00	0.12	1.00	1.00	1.00	1.00	**0.04**	1.00	1.00
IL	1.00	1.00	1.00	1.00	1.00	**0.05**	1.00	1.00	0.32	1.00	1.00	1.00	1.00	0.30	1.00	1.00	1.00	1.00	1.00	1.00	1.00	0.09	1.00	1.00	0.17	1.00	1.00
MSL	1.00	1.00	1.00	1.00	**0.02**	1.00	1.00	1.00	1.00	1.00	1.00	1.00	1.00	1.00	1.00	1.00	1.00	1.00	1.00	0.15	1.00	1.00	1.00	1.00	1.00	1.00	1.00
MSW	1.00	1.00	0.18	**0.00**	0.06	1.00	**0.01**	**0.03**	1.00	0.52	1.00	**0.00**	**0.00**	1.00	**0.03**	**0.00**	1.00	**0.00**	**0.00**	0.88	0.40	1.00	1.00	1.00	1.00	1.00	1.00
FSL	1.00	1.00	1.00	**0.03**	1.00	1.00	1.00	1.00	1.00	1.00	1.00	1.00	1.00	0.17	1.00	1.00	1.00	1.00	1.00	1.00	1.00	**0.00**	1.00	1.00	1.00	1.00	1.00
FSW	1.00	0.20	0.95	1.00	1.00	1.00	1.00	1.00	1.00	1.00	**0.01**	1.00	1.00	1.00	1.00	1.00	1.00	**0.02**	1.00	1.00	1.00	0.22	**0.02**	1.00	1.00	**0.05**	1.00
PL	0.27	**0.00**	1.00	1.00	1.00	1.00	1.00	1.00	0.51	1.00	1.00	1.00	1.00	1.00	1.00	1.00	1.00	1.00	1.00	1.00	1.00	1.00	1.00	1.00	1.00	1.00	**0.00**
BL	1.00	1.00	1.00	1.00	1.00	1.00	1.00	1.00	1.00	1.00	1.00	1.00	1.00	1.00	1.00	1.00	1.00	1.00	1.00	1.00	1.00	0.74	1.00	1.00	1.00	1.00	1.00
UL	1.00	**0.00**	**0.00**	1.00	1.00	1.00	**0.01**	1.00	1.00	1.00	1.00	0.93	**0.00**	1.00	1.00	1.00	1.00	**0.00**	1.00	1.00	1.00	**0.00**	**0.05**	1.00	1.00	**0.00**	1.00
UBL	0.35	0.24	1.00	1.00	1.00	1.00	0.79	1.00	1.00	1.00	1.00	1.00	1.00	1.00	1.00	1.00	1.00	**0.00**	0.14	1.00	1.00	1.00	1.00	1.00	1.00	0.63	1.00
UBL/UL	**0.00**	1.00	1.00	1.00	1.00	1.00	1.00	1.00	1.00	1.00	1.00	1.00	0.08	1.00	1.00	1.00	1.00	**0.00**	0.10	1.00	1.00	1.00	1.00	0.23	1.00	1.00	1.00
GL	**0.00**	**0.00**	**0.00**	0.32	1.00	**0.00**	**0.00**	0.07	0.08	**0.00**	1.00	**0.01**	**0.00**	1.00	0.20	**0.00**	**0.00**	**0.00**	**0.00**	1.00	1.00	1.00	1.00	1.00	1.00	1.00	1.00

Traits	Dunn's multiple comparisons test																										
	8-15	8-16	8-17	8-18	8-19	8-20	8-21	8-22	8-23	8-24	8-25	8-26	9-10	9-11	9-12	9-13	9-14	9-15	9-16	9-17	9-18	9-19	9-20	9-21	9-22	9-23	9-24
	p	*p*	*p*	*p*	*p*	*p*	*p*	*p*	*p*	*p*	*p*	*p*	*p*	*p*	*p*	*p*	*p*	*p*	*p*	*p*	*p*	*p*	*p*	*p*	*p*	*p*	*p*
CH	**0.00**	1.00	1.00	1.00	1.00	1.00	1.00	0.22	1.00	1.00	**0.02**	1.00	1.00	**0.00**	1.00	**0.02**	1.00	1.00	0.87	1.00	1.00	1.00	1.00	1.00	1.00	**0.00**	1.00
CLL	1.00	1.00	1.00	0.11	1.00	0.17	**0.00**	1.00	1.00	1.00	1.00	**0.01**	1.00	**0.00**	1.00	**0.01**	1.00	1.00	0.11	0.50	1.00	1.00	1.00	1.00	1.00	**0.00**	1.00
CLW	1.00	1.00	1.00	1.00	1.00	0.86	**0.03**	1.00	1.00	1.00	1.00	1.00	1.00	1.00	1.00	1.00	1.00	1.00	1.00	1.00	1.00	1.00	1.00	**0.04**	1.00	1.00	1.00
NFS	1.00	1.00	1.00	1.00	1.00	1.00	1.00	0.64	1.00	1.00	1.00	1.00	1.00	1.00	1.00	1.00	1.00	0.82	1.00	0.61	1.00	1.00	1.00	**0.00**	1.00	1.00	1.00
NMS	1.00	1.00	1.00	1.00	1.00	1.00	1.00	1.00	1.00	1.00	1.00	1.00	1.00	1.00	**0.00**	1.00	1.00	1.00	1.00	1.00	1.00	1.00	1.00	1.00	1.00	1.00	1.00
IL	1.00	1.00	1.00	0.87	1.00	1.00	1.00	1.00	0.24	1.00	1.00	1.00	1.00	**0.00**	1.00	1.00	**0.00**	1.00	1.00	1.00	1.00	**0.00**	0.38	1.00	**0.01**	1.00	1.00
MSL	1.00	1.00	1.00	1.00	0.26	1.00	1.00	1.00	1.00	1.00	1.00	1.00	1.00	1.00	1.00	1.00	1.00	1.00	1.00	1.00	1.00	0.26	1.00	1.00	1.00	1.00	1.00
MSW	1.00	1.00	1.00	1.00	1.00	1.00	1.00	1.00	1.00	1.00	1.00	1.00	1.00	1.00	1.00	1.00	**0.05**	1.00	**0.01**	1.00	1.00	**0.02**	1.00	1.00	**0.00**	1.00	1.00
FSL	1.00	1.00	1.00	1.00	**0.01**	1.00	1.00	1.00	1.00	1.00	1.00	0.09	**0.00**	1.00	**0.00**	1.00	0.46	**0.00**	**0.00**	0.31	1.00	1.00	**0.00**	**0.00**	**0.00**	**0.02**	**0.00**
FSW	1.00	1.00	1.00	1.00	1.00	0.11	**0.00**	1.00	1.00	1.00	1.00	0.09	1.00	1.00	1.00	1.00	1.00	1.00	**0.00**	0.34	0.63	1.00	1.00	1.00	0.55	**0.00**	1.00
PL	1.00	1.00	1.00	0.65	1.00	**0.00**	0.60	1.00	**0.02**	1.00	0.48	1.00	1.00	1.00	1.00	1.00	1.00	1.00	1.00	1.00	1.00	1.00	1.00	1.00	1.00	1.00	1.00
BL	1.00	1.00	1.00	1.00	1.00	1.00	1.00	1.00	1.00	1.00	1.00	1.00	1.00	**0.00**	1.00	1.00	**0.05**	1.00	1.00	1.00	1.00	**0.00**	1.00	1.00	**0.00**	1.00	0.09
UL	1.00	1.00	1.00	1.00	0.78	**0.02**	**0.00**	**0.00**	1.00	0.12	**0.01**	**0.00**	1.00	1.00	**0.00**	1.00	1.00	0.41	1.00	0.13	**0.00**	1.00	1.00	1.00	1.00	**0.00**	1.00
UBL	1.00	1.00	1.00	1.00	1.00	1.00	1.00	1.00	0.75	1.00	1.00	**0.00**	1.00	0.14	0.09	1.00	1.00	0.15	1.00	1.00	1.00	1.00	1.00	1.00	1.00	**0.00**	**0.01**
UBL/UL	0.14	1.00	1.00	1.00	1.00	1.00	0.13	0.16	**0.00**	**0.00**	**0.00**	1.00	1.00	1.00	1.00	1.00	1.00	1.00	1.00	1.00	0.74	1.00	1.00	1.00	1.00	**0.00**	**0.02**
GL	1.00	0.36	1.00	1.00	**0.00**	1.00	1.00	1.00	1.00	1.00	**0.00**	0.21	1.00	1.00	1.00	1.00	1.00	1.00	1.00	1.00	1.00	1.00	1.00	1.00	0.21	0.16	1.00

Traits	Dunn's multiple comparisons test																										
	9-25	9-26	10-11	10-12	10-13	10-14	10-15	10-16	10-17	10-18	10-19	10-20	10-21	10-22	10-23	10-24	10-25	10-26	11-12	11-13	11-14	11-15	11-16	11-17	11-18	11-19	11-20
	p	*p*	*p*	*p*	*p*	*p*	*p*	*p*	*p*	*p*	*p*	*p*	*p*	*p*	*p*	*p*	*p*	*p*	*p*	*p*	*p*	*p*	*p*	*p*	*p*	*p*	*p*
CH	1.00	1.00	**0.00**	1.00	**0.00**	1.00	1.00	**0.01**	1.00	0.20	**0.01**	1.00	1.00	1.00	**0.00**	1.00	1.00	1.00	**0.00**	1.00	**0.00**	**0.00**	1.00	**0.00**	0.24	1.00	**0.02**
CLL	0.11	1.00	**0.02**	1.00	0.23	1.00	1.00	1.00	1.00	1.00	1.00	1.00	1.00	1.00	**0.00**	1.00	1.00	1.00	**0.00**	1.00	0.13	0.41	1.00	1.00	**0.00**	0.06	**0.00**
CLW	1.00	1.00	1.00	1.00	1.00	1.00	1.00	1.00	1.00	0.09	1.00	**0.01**	**0.00**	**0.01**	1.00	0.15	1.00	**0.04**	1.00	1.00	1.00	1.00	1.00	0.18	**0.01**	0.60	**0.00**
NFS	**0.00**	0.81	1.00	0.30	1.00	1.00	1.00	1.00	1.00	1.00	1.00	1.00	1.00	1.00	1.00	1.00	1.00	1.00	1.00	1.00	1.00	1.00	1.00	1.00	1.00	1.00	1.00
NMS	1.00	1.00	1.00	0.28	1.00	1.00	1.00	1.00	1.00	1.00	1.00	1.00	1.00	1.00	1.00	1.00	1.00	1.00	1.00	1.00	1.00	1.00	1.00	0.44	1.00	1.00	1.00
IL	1.00	1.00	**0.01**	1.00	1.00	0.06	1.00	1.00	1.00	1.00	**0.03**	1.00	1.00	0.42	1.00	1.00	1.00	1.00	**0.00**	1.00	1.00	**0.01**	1.00	0.15	**0.00**	1.00	1.00
MSL	1.00	1.00	**0.02**	1.00	**0.00**	0.10	1.00	0.41	**0.01**	1.00	**0.00**	0.42	0.13	1.00	0.22	1.00	1.00	**0.01**	0.84	1.00	1.00	1.00	1.00	1.00	1.00	1.00	1.00
MSW	0.39	1.00	1.00	1.00	1.00	1.00	1.00	0.76	1.00	1.00	1.00	1.00	1.00	0.30	1.00	1.00	1.00	1.00	1.00	1.00	1.00	1.00	1.00	1.00	1.00	1.00	1.00
FSL	**0.00**	1.00	**0.00**	1.00	0.19	1.00	1.00	1.00	**0.00**	**0.00**	**0.00**	1.00	1.00	1.00	0.17	1.00	1.00	**0.00**	1.00	1.00	1.00	**0.05**	1.00	1.00	1.00	1.00	**0.00**
FSW	1.00	1.00	1.00	1.00	1.00	1.00	1.00	**0.00**	**0.01**	**0.03**	1.00	1.00	1.00	**0.03**	**0.00**	1.00	0.42	1.00	1.00	1.00	1.00	1.00	**0.02**	1.00	1.00	1.00	1.00
PL	1.00	1.00	1.00	1.00	1.00	**0.00**	1.00	1.00	1.00	1.00	1.00	**0.00**	1.00	1.00	0.10	1.00	1.00	1.00	1.00	1.00	**0.00**	1.00	1.00	1.00	1.00	1.00	**0.00**
BL	1.00	1.00	1.00	1.00	1.00	1.00	1.00	1.00	1.00	1.00	1.00	1.00	1.00	1.00	1.00	1.00	1.00	1.00	**0.01**	1.00	1.00	0.27	1.00	0.91	**0.00**	1.00	0.75
UL	1.00	1.00	1.00	0.11	1.00	1.00	1.00	1.00	1.00	0.06	1.00	1.00	1.00	1.00	**0.00**	1.00	1.00	1.00	1.00	1.00	1.00	1.00	1.00	1.00	1.00	1.00	1.00
UBL	0.19	1.00	0.81	0.55	1.00	1.00	0.93	1.00	1.00	1.00	1.00	1.00	1.00	1.00	**0.00**	0.10	1.00	1.00	1.00	**0.00**	0.32	1.00	1.00	1.00	**0.01**	0.65	1.00
UBL/UL	0.45	1.00	1.00	1.00	1.00	1.00	1.00	1.00	1.00	1.00	1.00	1.00	1.00	1.00	**0.00**	**0.01**	0.19	1.00	1.00	0.08	0.15	1.00	1.00	1.00	**0.00**	0.37	1.00
GL	1.00	1.00	1.00	1.00	1.00	1.00	0.44	1.00	1.00	1.00	1.00	1.00	1.00	**0.03**	**0.02**	1.00	1.00	1.00	1.00	1.00	1.00	1.00	1.00	1.00	1.00	**0.02**	1.00

Traits	Dunn's multiple comparisons test																										
	11-21	11-22	11-23	11-24	11-25	11-26	12-13	12-14	12-15	12-16	12-17	12-18	12-19	12-20	12-21	12-22	12-23	12-24	12-25	12-26	13-14	13-15	13-16	13-17	13-18	13-19	13-20
	p	*p*	*p*	*p*	*p*	*p*	*p*	*p*	*p*	*p*	*p*	*p*	*p*	*p*	*p*	*p*	*p*	*p*	*p*	*p*	*p*	*p*	*p*	*p*	*p*	*p*	*p*
CH	**0.00**	**0.00**	1.00	**0.01**	**0.00**	**0.00**	**0.00**	1.00	1.00	**0.03**	1.00	0.55	**0.04**	1.00	1.00	1.00	**0.00**	1.00	1.00	1.00	**0.00**	**0.00**	1.00	**0.03**	1.00	1.00	0.33
CLL	**0.00**	0.59	1.00	1.00	1.00	**0.00**	**0.00**	1.00	1.00	**0.00**	**0.00**	1.00	0.59	1.00	1.00	0.07	**0.00**	**0.04**	**0.00**	1.00	0.94	1.00	1.00	1.00	**0.01**	0.75	**0.02**
CLW	**0.00**	**0.00**	1.00	**0.02**	0.73	**0.00**	1.00	1.00	1.00	1.00	1.00	1.00	1.00	1.00	0.71	1.00	1.00	1.00	1.00	1.00	1.00	1.00	0.10	0.34	**0.02**	1.00	**0.00**
NFS	1.00	1.00	1.00	1.00	1.00	1.00	0.30	1.00	**0.03**	1.00	**0.01**	0.29	0.89	1.00	**0.00**	1.00	**0.04**	**0.04**	**0.00**	**0.02**	1.00	1.00	1.00	1.00	1.00	1.00	1.00
NMS	1.00	1.00	1.00	1.00	1.00	1.00	**0.03**	1.00	1.00	1.00	**0.00**	1.00	0.06	0.23	**0.01**	**0.01**	0.18	0.38	**0.01**	1.00	1.00	1.00	1.00	1.00	1.00	1.00	1.00
IL	0.08	1.00	**0.00**	0.53	**0.03**	0.10	1.00	**0.00**	1.00	1.00	1.00	1.00	**0.00**	0.78	1.00	**0.01**	1.00	1.00	1.00	1.00	1.00	1.00	1.00	1.00	1.00	1.00	1.00
MSL	1.00	1.00	1.00	1.00	0.10	1.00	0.19	1.00	1.00	1.00	1.00	1.00	**0.00**	1.00	1.00	1.00	1.00	1.00	1.00	1.00	1.00	1.00	1.00	1.00	1.00	1.00	1.00
MSW	**0.02**	1.00	1.00	1.00	1.00	1.00	1.00	1.00	1.00	0.14	1.00	1.00	1.00	1.00	1.00	**0.03**	1.00	1.00	1.00	1.00	1.00	1.00	0.55	1.00	1.00	1.00	1.00
FSL	0.36	0.10	1.00	**0.00**	1.00	1.00	1.00	1.00	1.00	1.00	1.00	0.85	**0.00**	1.00	1.00	1.00	1.00	1.00	1.00	0.06	1.00	1.00	1.00	1.00	1.00	1.00	**0.04**
FSW	1.00	1.00	0.06	1.00	1.00	1.00	1.00	1.00	1.00	0.90	1.00	1.00	1.00	1.00	1.00	1.00	1.00	1.00	1.00	1.00	1.00	1.00	**0.00**	0.06	0.11	1.00	1.00
PL	1.00	1.00	0.10	1.00	1.00	1.00	1.00	1.00	1.00	1.00	1.00	1.00	1.00	1.00	1.00	1.00	1.00	1.00	1.00	1.00	1.00	1.00	1.00	1.00	1.00	1.00	1.00
BL	**0.05**	1.00	0.95	1.00	0.27	0.21	1.00	0.15	1.00	1.00	1.00	1.00	**0.00**	1.00	1.00	**0.01**	1.00	0.31	1.00	1.00	1.00	1.00	1.00	1.00	1.00	1.00	1.00
UL	**0.01**	0.60	1.00	1.00	1.00	**0.00**	**0.01**	1.00	1.00	1.00	1.00	1.00	1.00	0.06	**0.00**	**0.00**	1.00	0.28	**0.01**	**0.00**	1.00	1.00	1.00	1.00	**0.00**	1.00	1.00
UBL	0.42	1.00	1.00	1.00	1.00	**0.00**	**0.00**	0.21	1.00	1.00	1.00	**0.00**	0.39	1.00	0.24	1.00	1.00	1.00	1.00	**0.00**	1.00	**0.01**	1.00	0.35	1.00	1.00	1.00
UBL/UL	1.00	1.00	1.00	1.00	1.00	**0.02**	1.00	1.00	1.00	1.00	1.00	0.06	1.00	1.00	1.00	1.00	**0.01**	0.25	1.00	1.00	1.00	**0.04**	1.00	1.00	1.00	1.00	1.00
GL	1.00	1.00	1.00	1.00	**0.00**	1.00	1.00	1.00	1.00	0.31	1.00	1.00	**0.00**	1.00	1.00	1.00	1.00	1.00	**0.00**	0.16	1.00	1.00	1.00	1.00	1.00	1.00	1.00

Traits	Dunn's multiple comparisons test																									
	13-21	13-22	13-23	13-24	13-25	13-26	14-15	14-16	14-17	14-18	14-19	14-20	14-21	14-22	14-23	14-24	14-25	14-26	15-16	15-17	15-18	15-19	15-20	15-21	15-22	15-23
	p	*p*	*p*	*p*	*p*	*p*	*p*	*p*	*p*	*p*	*p*	*p*	*p*	*p*	*p*	*p*	*p*	*p*	*p*	*p*	*p*	*p*	*p*	*p*	*p*	*p*
CH	**0.02**	**0.00**	1.00	0.20	**0.00**	**0.01**	1.00	0.11	1.00	1.00	0.18	1.00	1.00	1.00	**0.00**	1.00	1.00	1.00	1.00	**0.00**	0.94	**0.01**	**0.00**	0.15	1.00	1.00
CLL	**0.00**	1.00	1.00	1.00	1.00	**0.00**	1.00	1.00	1.00	1.00	1.00	1.00	1.00	1.00	**0.00**	1.00	1.00	1.00	1.00	1.00	1.00	1.00	1.00	1.00	1.00	**0.01**
CLW	**0.00**	**0.00**	1.00	**0.04**	1.00	**0.01**	1.00	1.00	1.00	1.00	1.00	1.00	0.06	1.00	1.00	1.00	1.00	1.00	1.00	1.00	1.00	1.00	0.10	0.32	1.00	1.00
NFS	1.00	1.00	1.00	1.00	1.00	1.00	1.00	1.00	1.00	1.00	1.00	1.00	0.13	1.00	1.00	1.00	0.30	1.00	1.00	1.00	1.00	1.00	1.00	1.00	1.00	1.00
NMS	1.00	1.00	1.00	1.00	1.00	1.00	1.00	1.00	1.00	1.00	1.00	1.00	1.00	1.00	1.00	1.00	1.00	1.00	1.00	1.00	1.00	1.00	1.00	1.00	1.00	1.00
IL	1.00	1.00	1.00	1.00	1.00	1.00	0.07	1.00	1.00	**0.01**	1.00	1.00	0.65	1.00	**0.00**	1.00	0.27	0.71	1.00	1.00	1.00	**0.04**	1.00	1.00	0.54	1.00
MSL	1.00	0.23	1.00	1.00	**0.01**	1.00	1.00	1.00	1.00	1.00	1.00	1.00	1.00	1.00	1.00	1.00	0.64	1.00	1.00	1.00	1.00	**0.01**	1.00	1.00	1.00	1.00
MSW	1.00	0.19	1.00	1.00	1.00	1.00	1.00	1.00	0.84	0.06	1.00	1.00	**0.00**	1.00	0.13	0.90	1.00	1.00	1.00	1.00	1.00	1.00	1.00	0.08	1.00	1.00
FSL	1.00	1.00	1.00	0.06	1.00	1.00	1.00	1.00	1.00	1.00	1.00	0.61	1.00	1.00	1.00	0.83	1.00	1.00	1.00	0.14	**0.01**	**0.00**	1.00	1.00	1.00	1.00
FSW	1.00	0.10	**0.00**	1.00	1.00	1.00	1.00	1.00	1.00	1.00	1.00	1.00	0.32	1.00	1.00	1.00	1.00	1.00	0.62	1.00	1.00	1.00	1.00	1.00	1.00	1.00
PL	1.00	1.00	1.00	1.00	1.00	1.00	**0.00**	**0.03**	**0.00**	0.87	**0.03**	1.00	0.78	0.28	1.00	**0.01**	1.00	**0.00**	1.00	1.00	1.00	1.00	**0.01**	1.00	1.00	0.45
BL	1.00	1.00	1.00	1.00	1.00	1.00	1.00	1.00	1.00	0.13	1.00	1.00	1.00	1.00	1.00	1.00	1.00	1.00	1.00	1.00	1.00	0.31	1.00	1.00	0.50	1.00
UL	1.00	1.00	**0.00**	1.00	1.00	1.00	1.00	1.00	1.00	1.00	1.00	1.00	0.06	1.00	1.00	1.00	1.00	**0.01**	1.00	1.00	1.00	1.00	1.00	**0.00**	0.13	1.00
UBL	1.00	1.00	**0.00**	**0.00**	**0.00**	1.00	0.36	1.00	1.00	1.00	1.00	1.00	1.00	1.00	**0.00**	**0.04**	0.51	1.00	1.00	1.00	**0.01**	0.66	1.00	0.41	1.00	1.00
UBL/UL	**0.03**	**0.04**	**0.00**	**0.00**	**0.00**	1.00	0.09	1.00	1.00	1.00	1.00	1.00	0.08	0.10	**0.00**	**0.00**	**0.00**	1.00	1.00	1.00	**0.00**	0.17	1.00	1.00	1.00	1.00
GL	1.00	1.00	0.83	1.00	0.45	1.00	1.00	1.00	1.00	1.00	1.00	1.00	1.00	1.00	1.00	1.00	0.50	1.00	**0.01**	0.88	1.00	**0.00**	0.09	1.00	1.00	1.00

Traits	Dunn's multiple comparisons test																										
	15-24	15-25	15-26	16-17	16-18	16-19	16-20	16-21	16-22	16-23	16-24	16-25	16-26	17-18	17-19	17-20	17-21	17-22	17-23	17-24	17-25	17-26	18-19	18-20	18-21	18-22	18-23
	p	*p*	*p*	*p*	*p*	*p*	*p*	*p*	*p*	*p*	*p*	*p*	*p*	*p*	*p*	*p*	*p*	*p*	*p*	*p*	*p*	*p*	*p*	*p*	*p*	*p*	*p*
CH	0.68	1.00	1.00	1.00	1.00	1.00	1.00	1.00	0.09	1.00	1.00	**0.01**	0.52	1.00	1.00	1.00	1.00	1.00	**0.00**	1.00	1.00	1.00	1.00	1.00	1.00	1.00	0.78
CLL	1.00	1.00	1.00	1.00	0.12	1.00	0.19	**0.00**	1.00	1.00	1.00	1.00	**0.01**	0.43	1.00	0.75	**0.00**	1.00	0.19	1.00	1.00	**0.04**	1.00	1.00	1.00	1.00	**0.00**
CLW	1.00	1.00	1.00	1.00	1.00	1.00	0.14	**0.00**	0.20	1.00	1.00	1.00	0.57	1.00	1.00	1.00	0.74	1.00	1.00	1.00	1.00	1.00	1.00	1.00	1.00	1.00	0.52
NFS	1.00	1.00	1.00	1.00	1.00	1.00	1.00	0.09	1.00	1.00	1.00	0.22	1.00	1.00	1.00	1.00	1.00	0.86	1.00	1.00	1.00	1.00	1.00	1.00	0.41	1.00	1.00
NMS	1.00	1.00	1.00	0.44	1.00	1.00	1.00	1.00	1.00	1.00	1.00	1.00	1.00	0.50	1.00	1.00	1.00	1.00	1.00	1.00	1.00	0.07	1.00	1.00	1.00	1.00	1.00
IL	1.00	1.00	1.00	1.00	1.00	1.00	1.00	1.00	1.00	1.00	1.00	1.00	1.00	1.00	0.83	1.00	1.00	1.00	1.00	1.00	1.00	1.00	**0.00**	1.00	1.00	**0.04**	1.00
MSL	1.00	1.00	1.00	1.00	1.00	0.63	1.00	1.00	1.00	1.00	1.00	1.00	1.00	1.00	0.13	1.00	1.00	1.00	1.00	1.00	**0.03**	1.00	**0.00**	1.00	1.00	1.00	1.00
MSW	1.00	1.00	1.00	**0.02**	**0.00**	1.00	0.74	**0.00**	1.00	**0.00**	**0.03**	1.00	1.00	1.00	0.42	1.00	1.00	**0.00**	1.00	1.00	1.00	1.00	**0.01**	1.00	1.00	**0.00**	1.00
FSL	1.00	1.00	**0.00**	1.00	1.00	**0.00**	1.00	1.00	1.00	1.00	1.00	1.00	0.09	1.00	1.00	**0.00**	1.00	0.23	1.00	**0.00**	1.00	1.00	1.00	**0.00**	0.06	**0.01**	1.00
FSW	1.00	1.00	1.00	1.00	1.00	**0.02**	**0.00**	**0.00**	1.00	1.00	**0.01**	1.00	**0.00**	1.00	1.00	0.08	**0.00**	1.00	1.00	1.00	1.00	0.07	1.00	0.19	**0.00**	1.00	1.00
PL	1.00	1.00	1.00	1.00	1.00	1.00	0.09	1.00	1.00	1.00	1.00	1.00	1.00	1.00	1.00	**0.00**	1.00	1.00	0.22	1.00	1.00	1.00	1.00	1.00	1.00	1.00	1.00
BL	1.00	1.00	1.00	1.00	1.00	1.00	1.00	1.00	1.00	1.00	1.00	1.00	1.00	1.00	0.86	1.00	1.00	1.00	1.00	1.00	1.00	1.00	**0.00**	1.00	1.00	**0.00**	1.00
UL	1.00	1.00	**0.00**	1.00	1.00	1.00	1.00	**0.00**	0.57	1.00	1.00	1.00	**0.00**	1.00	1.00	1.00	**0.00**	**0.01**	0.34	1.00	1.00	**0.00**	1.00	**0.01**	**0.00**	**0.00**	1.00
UBL	1.00	1.00	**0.00**	1.00	1.00	1.00	1.00	1.00	1.00	**0.01**	1.00	1.00	0.06	0.59	1.00	1.00	1.00	1.00	**0.00**	1.00	1.00	**0.00**	1.00	1.00	1.00	1.00	**0.00**
UBL/UL	1.00	1.00	**0.01**	1.00	1.00	1.00	1.00	1.00	1.00	**0.00**	**0.00**	0.14	1.00	**0.00**	1.00	1.00	1.00	1.00	**0.00**	**0.00**	0.14	1.00	0.59	**0.00**	**0.00**	**0.00**	**0.00**
GL	1.00	**0.00**	**0.00**	1.00	1.00	1.00	1.00	**0.00**	**0.00**	**0.00**	0.09	1.00	1.00	1.00	0.19	1.00	0.36	**0.02**	**0.01**	1.00	**0.03**	1.00	0.06	1.00	1.00	0.11	0.08

Traits	Dunn's multiple comparisons test																								
	18-24	18-25	18-26	19-20	19-21	19-22	19-23	19-24	19-25	19-26	20-21	20-22	20-23	20-24	20-25	20-26	21-22	21-23	21-24	21-25	21-26	22-23	22-24	22-25	22-26	23-24
	p	*p*	*p*	*p*	*p*	*p*	*p*	*p*	*p*	*p*	*p*	*p*	*p*	*p*	*p*	*p*	*p*	*p*	*p*	*p*	*p*	*p*	*p*	*p*	*p*	*p*
CH	1.00	0.09	1.00	1.00	1.00	0.06	1.00	1.00	**0.00**	0.76	1.00	1.00	**0.03**	1.00	1.00	1.00	1.00	**0.00**	1.00	1.00	1.00	**0.00**	1.00	1.00	1.00	**0.02**
CLL	1.00	0.06	1.00	1.00	0.51	1.00	**0.00**	1.00	1.00	1.00	1.00	1.00	**0.00**	1.00	0.12	1.00	**0.03**	**0.00**	**0.02**	**0.00**	1.00	**0.00**	1.00	1.00	1.00	0.11
CLW	1.00	1.00	1.00	1.00	0.14	1.00	1.00	1.00	1.00	1.00	1.00	1.00	**0.03**	1.00	1.00	1.00	1.00	**0.00**	1.00	0.24	1.00	**0.05**	1.00	1.00	1.00	0.99
NFS	1.00	1.00	1.00	1.00	0.06	1.00	1.00	1.00	0.25	1.00	**0.01**	1.00	1.00	1.00	**0.03**	1.00	**0.00**	1.00	1.00	1.00	1.00	1.00	1.00	**0.00**	1.00	1.00
NMS	1.00	1.00	1.00	1.00	1.00	1.00	1.00	1.00	1.00	1.00	1.00	1.00	1.00	1.00	1.00	1.00	1.00	1.00	1.00	1.00	1.00	1.00	1.00	1.00	1.00	1.00
IL	1.00	1.00	1.00	1.00	0.38	1.00	**0.00**	1.00	0.13	0.55	1.00	1.00	0.91	1.00	1.00	1.00	1.00	1.00	1.00	1.00	1.00	**0.04**	1.00	1.00	1.00	1.00
MSL	1.00	1.00	1.00	**0.00**	**0.00**	**0.00**	**0.00**	**0.00**	**0.00**	0.53	1.00	1.00	1.00	1.00	1.00	1.00	1.00	1.00	1.00	0.82	1.00	1.00	1.00	1.00	1.00	1.00
MSW	1.00	0.33	1.00	1.00	**0.00**	1.00	**0.03**	0.64	1.00	1.00	**0.03**	0.13	1.00	1.00	1.00	1.00	**0.00**	1.00	1.00	**0.00**	**0.00**	**0.00**	**0.00**	1.00	1.00	1.00
FSL	**0.00**	1.00	1.00	**0.00**	**0.00**	**0.00**	0.08	**0.00**	**0.00**	1.00	1.00	1.00	**0.01**	1.00	0.27	**0.00**	1.00	1.00	1.00	1.00	**0.00**	1.00	1.00	1.00	**0.00**	**0.03**
FSW	1.00	1.00	0.17	1.00	1.00	1.00	**0.02**	1.00	1.00	1.00	1.00	0.16	**0.00**	1.00	1.00	1.00	**0.00**	**0.00**	1.00	**0.01**	1.00	1.00	1.00	1.00	0.14	**0.01**
PL	1.00	1.00	1.00	0.06	1.00	1.00	1.00	1.00	1.00	1.00	1.00	0.80	1.00	**0.01**	1.00	**0.00**	1.00	1.00	1.00	1.00	1.00	1.00	1.00	1.00	1.00	1.00
BL	0.18	1.00	1.00	0.74	**0.01**	1.00	0.94	1.00	0.19	0.15	1.00	1.00	1.00	1.00	1.00	1.00	**0.03**	1.00	1.00	1.00	1.00	1.00	1.00	0.35	0.27	1.00
UL	0.12	**0.00**	**0.00**	1.00	**0.00**	0.30	**0.01**	1.00	1.00	**0.00**	0.24	1.00	**0.00**	1.00	1.00	0.06	1.00	**0.00**	0.14	1.00	1.00	**0.00**	1.00	1.00	1.00	**0.00**
UBL	**0.00**	**0.00**	1.00	1.00	1.00	1.00	**0.00**	**0.02**	0.71	**0.04**	1.00	1.00	**0.00**	0.48	1.00	**0.00**	1.00	**0.00**	**0.01**	0.40	0.07	**0.00**	0.58	1.00	**0.00**	1.00
UBL/UL	**0.00**	**0.00**	1.00	1.00	0.08	0.11	**0.00**	**0.00**	**0.00**	1.00	1.00	1.00	**0.00**	**0.04**	1.00	0.86	1.00	0.08	1.00	1.00	**0.00**	0.07	1.00	1.00	**0.00**	1.00
GL	1.00	**0.01**	1.00	1.00	**0.00**	**0.00**	**0.00**	**0.00**	1.00	1.00	**0.01**	**0.00**	**0.00**	0.73	1.00	1.00	1.00	1.00	1.00	**0.00**	**0.00**	1.00	1.00	**0.00**	**0.00**	1.00

Traits	Dunn's multiple comparisons test				
	23-25	23-26	24-25	24-26	25-26
	p	*p*	*p*	*p*	*p*
CH	**0.00**	**0.00**	1.00	1.00	1.00
CLL	1.00	**0.00**	1.00	0.69	**0.01**
CLW	1.00	0.25	1.00	1.00	1.00
NFS	1.00	1.00	1.00	1.00	1.00
NMS	1.00	1.00	1.00	1.00	1.00
IL	1.00	1.00	1.00	1.00	1.00
MSL	1.00	1.00	1.00	1.00	0.06
MSW	0.96	1.00	1.00	1.00	1.00
FSL	1.00	1.00	0.50	**0.00**	0.21
FSW	1.00	**0.00**	1.00	1.00	1.00
PL	1.00	0.16	1.00	1.00	1.00
BL	1.00	1.00	1.00	1.00	1.00
UL	**0.00**	**0.00**	1.00	**0.03**	0.24
UBL	0.69	**0.00**	1.00	**0.00**	**0.00**
UBL/UL	1.00	**0.00**	1.00	**0.00**	**0.00**
GL	**0.00**	**0.00**	**0.00**	**0.02**	1.00

Supplementary Table S4

Morphological variability of *Carex buekii* (Cyperaceae) as a function of soil conditions. A case study of the Central European populations

Helena Więcław[1]. Beata Bosiacka[1]. Richard Hrivnák[2]. Zygmunt Dajdok[3]. Attila Mesterházy[4]. Jacob Koopman[5]
[1]University of Szczecin. Institute of Marine and Environmental Sciences. Adama Mickiewicza 18. PL-70-383. Szczecin. Poland; [2]Slovak Academy of Sciences. Institute of Botany. Plant Science and Biodiversity Center. Dúbravská cesta 9. SK-845 23 Bratislava. Slovakia; [3]University of Wrocław. Faculty of Biological Sciences. Department of Botany. Kanonia 6/8. PL-50-328. Wrocław. Poland; [4]Centre for Ecological Research. Wetland Ecology Research Group. Bem tér 18/C. H-4026 Debrecen. Hungary: [5]ul. Kochanowskiego 27. PL-73-200 Choszczno. Poland
e-mail: helena.wieclaw@usz.edu.pl

Factor loadings onto principal component axes for 16 traits used in the proncipal components analysis.
Values higher than 0.50 are set in boldface.
Explanations: CH – Culm height; CLL – Cauline leaf length; CLW – Cauline leaf width; NFS – Number of female spikes; NMS – Number of male spikes; IL – Inflorescence length; MSL – Male spike length; MSW – Male spike width; FSL – Female spike length; FSW – Female spike width; PL – Peduncle length; BL – Bract length; UL – Utricle length; UBL – Utricle beak length; UBL/UL – Ratio of beak length to utricle length; GL – Glume length.

Traits	PCA 1	PCA 2	PCA 3	PCA 4	PCA 5	PCA 6	PCA 7	PCA 8	PCA 9	PCA 10	PCA 11	PCA 12	PCA 13	PCA 14	PCA 15	PCA 16
CH [cm]	**-0.818**	0.163	-0.163	0.020	-0.268	-0.059	-0.165	0.251	-0.124	-0.281	-0.080	0.026	0.023	-0.083	0.058	-0.001
CLL [cm]	**-0.731**	0.474	-0.294	-0.015	0.018	0.045	-0.238	-0.167	0.064	-0.104	-0.098	0.114	-0.109	0.130	-0.038	0.001
CLW [cm]	**-0.725**	-0.012	-0.175	0.263	-0.264	0.027	-0.410	-0.065	0.237	0.235	0.052	-0.029	0.122	-0.027	0.011	0.000
NFS [no]	-0.439	-0.045	-0.446	**-0.641**	0.033	0.180	0.012	-0.108	-0.285	0.165	0.115	0.161	-0.003	-0.037	0.009	0.000
NMS [no]	0.229	-0.365	-0.246	**0.740**	-0.087	0.000	-0.032	0.351	-0.162	0.034	0.145	0.153	-0.021	0.047	-0.021	0.000
IL [cm]	**-0.828**	0.235	0.006	-0.057	0.340	-0.043	0.192	0.226	0.010	0.067	0.118	-0.130	0.004	0.099	0.079	-0.006
MSL [cm]	**-0.611**	0.406	0.444	0.057	-0.190	-0.161	0.313	0.099	-0.114	0.119	-0.173	0.102	0.148	0.023	-0.040	0.003
MSW [cm]	0.430	0.006	-0.258	**-0.554**	**-0.571**	0.027	-0.062	0.272	-0.017	-0.039	0.055	-0.148	0.059	0.072	-0.046	0.001
FSL [cm]	-0.435	0.243	**0.506**	0.039	**-0.602**	-0.148	0.166	-0.123	0.054	0.018	0.193	0.017	-0.164	-0.024	0.004	-0.001
FSW [cm]	-0.174	**-0.797**	-0.119	-0.243	-0.087	-0.317	0.009	0.222	0.106	0.165	-0.207	0.028	-0.144	-0.002	0.019	0.000
PL [cm]	0.180	0.105	**0.503**	-0.208	0.263	**-0.611**	-0.423	0.021	-0.173	0.007	0.083	0.004	0.015	0.012	-0.012	0.001
BL [cm]	**-0.847**	0.096	-0.251	0.025	0.354	-0.071	0.091	0.173	0.048	-0.010	0.066	-0.120	-0.055	-0.083	-0.102	0.005
UL [mm]	-0.399	**-0.750**	-0.166	-0.115	0.003	-0.308	0.177	-0.183	0.145	-0.167	0.097	0.083	0.108	0.021	-0.020	-0.020
UBL [mm]	-0.457	**-0.694**	0.446	-0.180	0.073	0.219	-0.026	0.020	0.073	-0.095	0.062	0.047	0.039	0.032	0.004	0.033
UBL/UL	-0.297	-0.290	**0.701**	-0.136	0.083	0.492	-0.191	0.169	-0.019	0.005	-0.023	0.014	-0.028	-0.003	-0.028	-0.025
GL [mm]	**-0.507**	**-0.594**	-0.026	0.323	-0.160	0.017	-0.023	-0.301	-0.344	0.014	-0.071	-0.207	-0.014	0.030	-0.007	0.000
Percentage of explained variance	30.64	17.51	12.32	10.09	7.68	5.96	4.22	3.83	2.43	1.61	1.31	1.13	0.74	0.29	0.14	0.10

Supplementary Table S5

Morphological variability of *Carex buekii* (Cyperaceae) as a function of soil conditions. A case study of the Central European populations

Helena Więcław[1], Beata Bosiacka[1], Richard Hrivnák[2], Zygmunt Dajdok[3], Attila Mesterházy[4], Jacob Koopman[5]

[1]University of Szczecin, Institute of Marine and Environmental Sciences, Adama Mickiewicza 18, PL-70-383, Szczecin, Poland; [2]Slovak Academy of Sciences, Institute of Botany, Plant Science and Biodiversity Center, Dúbravská cesta 9, SK-845 23 Bratislava, Slovakia; [3]University of Wrocław, Faculty of Biological Sciences, Department of Botany, Kanonia 6/8, PL-50-328, Wrocław, Poland; [4]Centre for Ecological Research, Wetland Ecology Research Group, Bem tér 18/C, H-4026 Debrecen, Hungary; [5]ul. Kochanowskiego 27, PL-73-200 Choszczno, Poland

e-mail: helena.wieclaw@usz.edu.pl

Results of Mann–Whitney *U* test, showing difference between two group of *C. buekii* populations (see Figure S1)

Explanations: *p* – significance level; significant differences ($p \leq 0.05$) have been marked with bold. CH – Culm height; CLL – Cauline leaf length; CLW – Cauline leaf width; NFS – Number of female spikes; NMS – Number of male spikes; IL – Inflorescence length; MSL – Male spike length; MSW – Male spike width; FSL – Female spike length; FSW – Female spike width; PL – Peduncle length; BL – Bract length; UL – Utricle length; UBL – Utricle beak length; UBL/UL – Ratio of beak length to utricle length; GL – Glume length.

**Traits**	**Mann–Whitney *U* test**				
	U	***Z***	***p***	***Z***	***p***
CH [cm]	11212.50	-10.796	**0.000**	-10.796	**0.000**
CLL [cm]	15522.00	-7.904	**0.000**	-7.904	**0.000**
CLW [cm]	8989.50	-12.288	**0.000**	-12.296	**0.000**
NFS [no]	24274.50	-2.030	**0.042**	-2.255	**0.024**
NMS [no]	25848.50	-0.974	0.330	-1.084	0.279
IL [cm]	17050.50	-6.878	**0.000**	-6.878	**0.000**
MSL [cm]	22516.50	-3,210	**0.001**	-3.214	**0.001**
MSW [cm]	19282.50	5.380	**0.000**	5.400	**0.000**
FSL [cm]	24582.00	-1.824	0.068	-1.824	0.068
FSW [cm]	23845.50	-2.318	**0.020**	-2.327	**0.020**
PL [cm]	26714.00	-0.393	0.694	-0.393	0.694
BL [cm]	17023.00	-6.897	**0.000**	-6.897	**0.000**
UL [mm]	22663.50	-3.111	**0.002**	-3.111	**0.002**
UBL [mm]	16125.00	-7.499	**0.000**	-7.513	**0.000**
UBL/UL	17610.00	-6.503	**0.000**	-6.522	**0.000**
GL [mm]	18769.50	-5.724	**0.000**	-5.25	**0.000**

Supplementary Figure S1

Helena Więcław[1], Beata Bosiacka[1], Richard Hrivnák[2], Zygmunt Dajdok[3], Attila Mesterházy[4], Jacob Koopman[5]

[1]University of Szczecin, Institute of Marine and Environmental Sciences, Adama Mickiewicza 18, PL-70-383, Szczecin, Poland; [2]Slovak Academy of Sciences, Institute of Botany, Plant Science and Biodiversity Center, Dúbravská cesta 9, SK-845 23 Bratislava, Slovakia; [3]University of Wrocław, Faculty of Biological Sciences, Department of Botany, Kanonia 6/8, PL-50-328, Wrocław, Poland; [4]Directorate of Hortobágy National Park, H-4024 Sumen utca 2, Debrecen, Hungary; [5]ul. Kochanowskiego 27, PL-73-200 Choszczno, Poland
e-mail: helena.wieclaw@usz.edu.pl

Ranges of morphological traits of two groups of *Carex buekii* distinguished on the basis of cluster analysis.
Large boxes indicate 25–75% of the interquartile ranges; small boxes – medians. Group I contains populations 8, 10, 12, 15, 17, 18, 19, 20, 21, 22, 23, 24, 25, and 26. Group II contains populations 1, 2, 3, 4, 5, 6, 7, 9, 11, 13, 14, and 16. The populations are numbered according to Table S1.

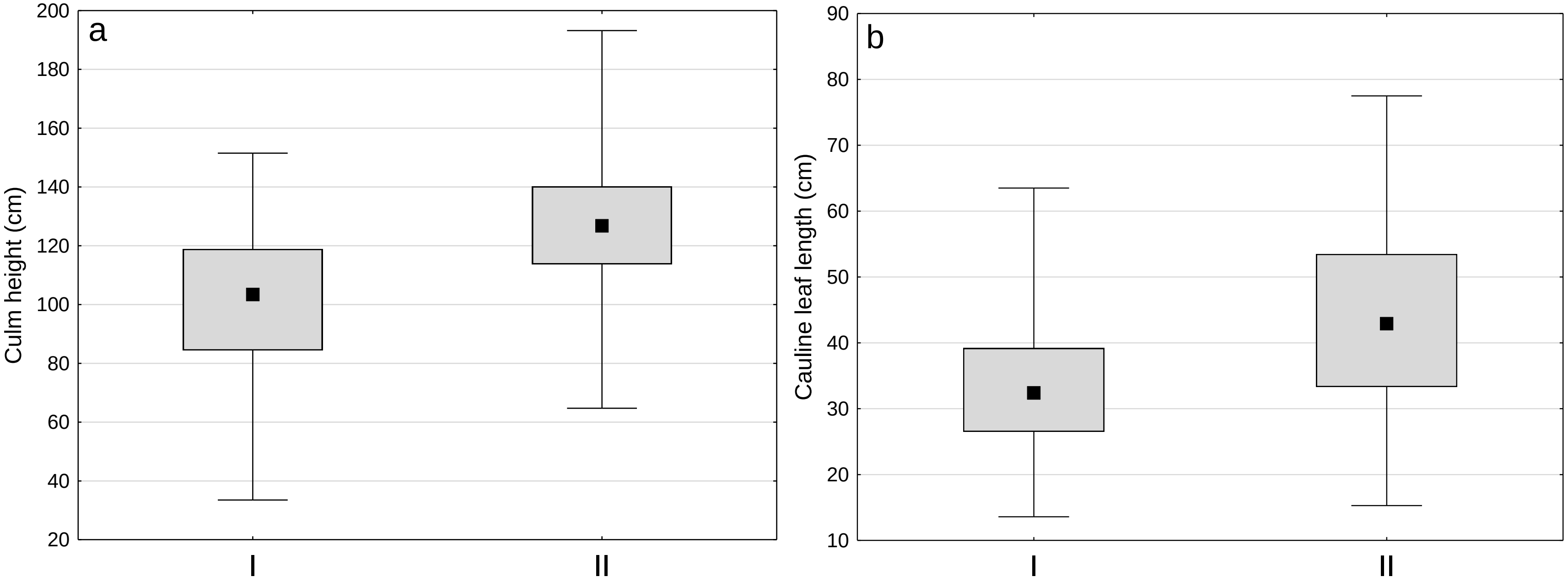

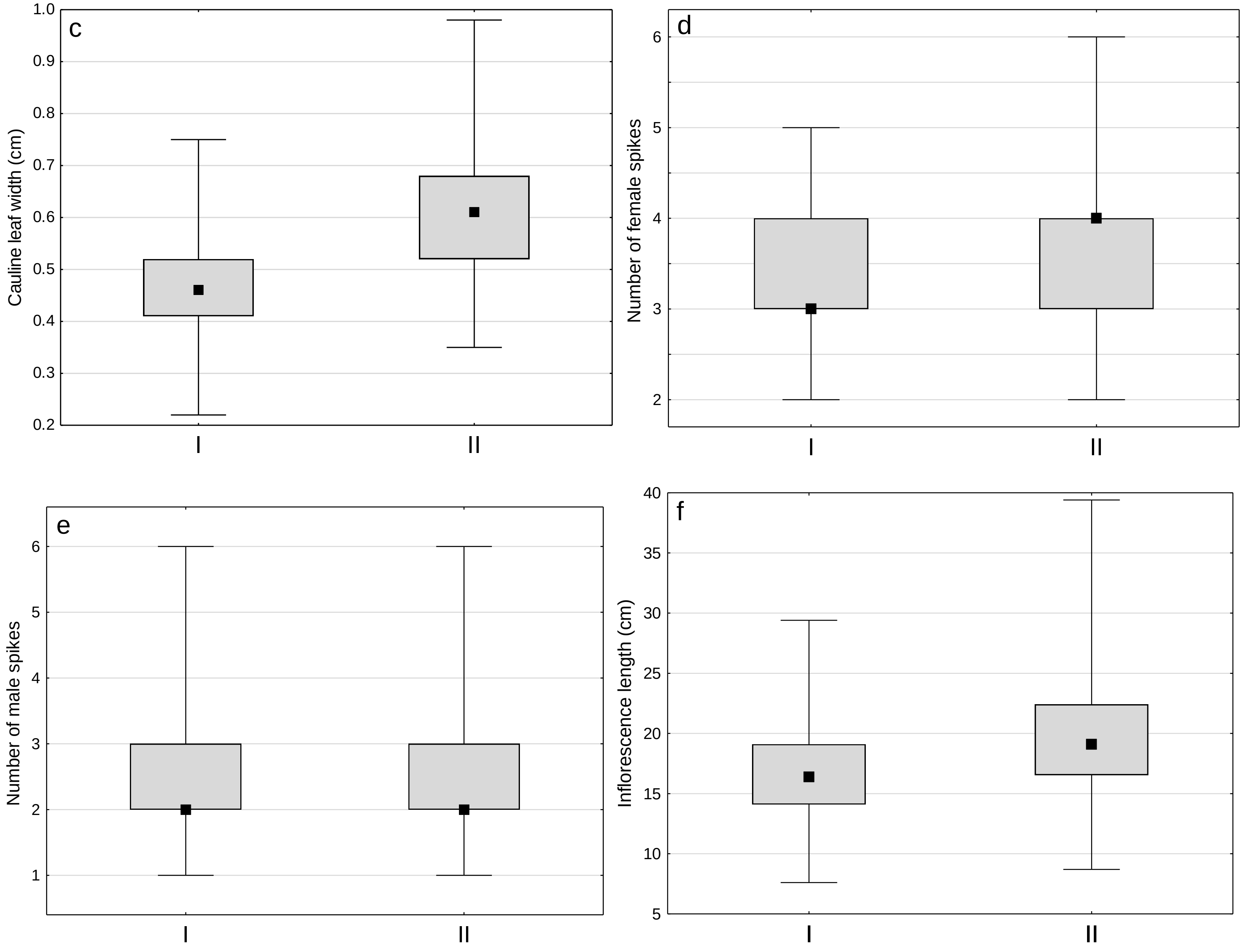

c
Cauline leaf width (cm)
1.0
0.9
0.8
0.7
0.6
0.5
0.4
0.3
0.2
I
II
d
Number of female spikes
6
5
4
3
2
I
II
e
Number of male spikes
6
5
4
3
2
1
I
II
f
Inflorescence length (cm)
40
35
30
25
20
15
10
5
I
II

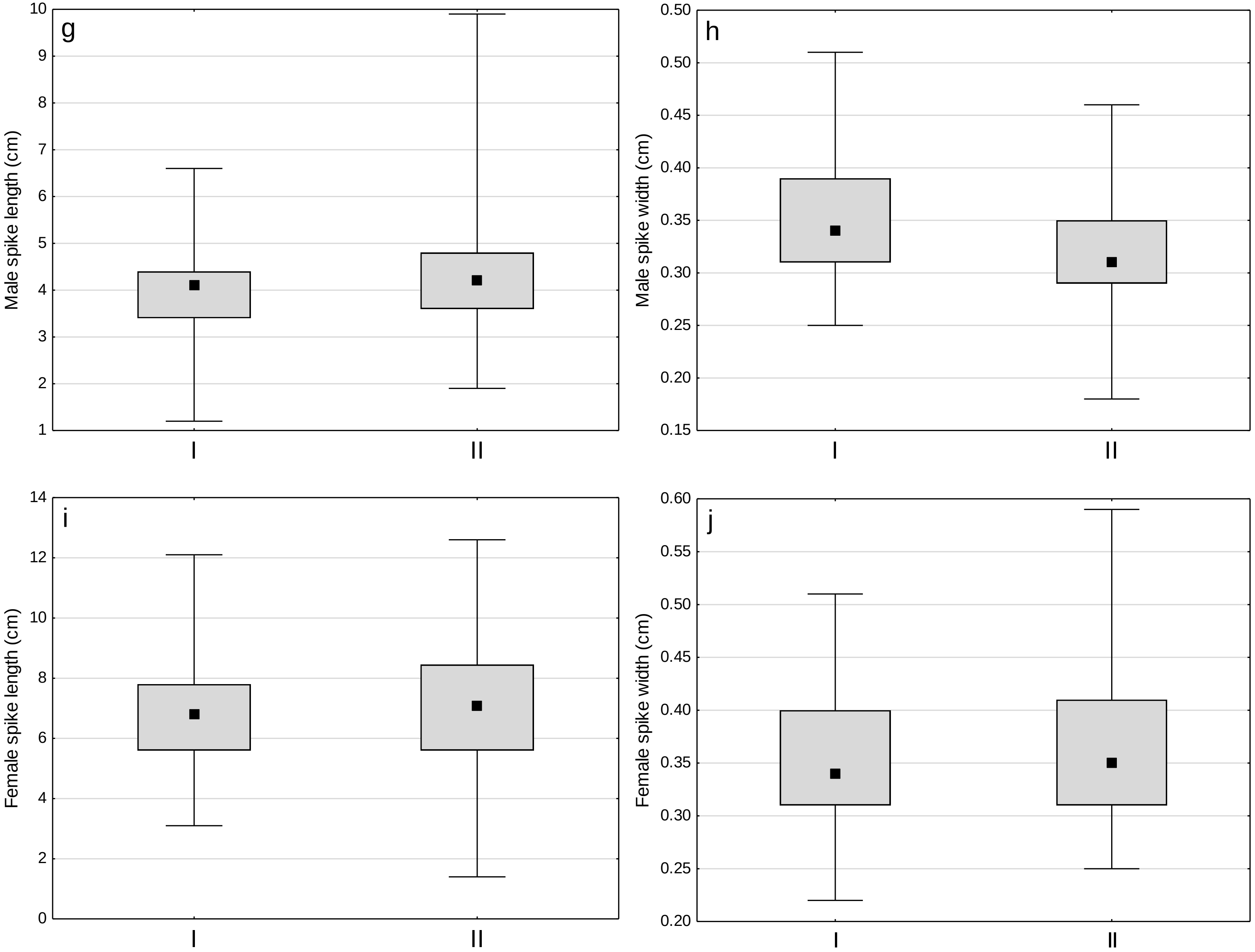

g
Male spike length (cm)
10
9
8
7
6
5
4
3
2
1
I
II
h
Male spike width (cm)
0.50
0.50
0.45
0.40
0.35
0.30
0.25
0.20
0.15
I
II
i
Female spike length (cm)
14
12
10
8
6
4
2
0
I
II
j
Female spike width (cm)
0.60
0.55
0.50
0.45
0.40
0.35
0.30
0.25
0.20
I
II

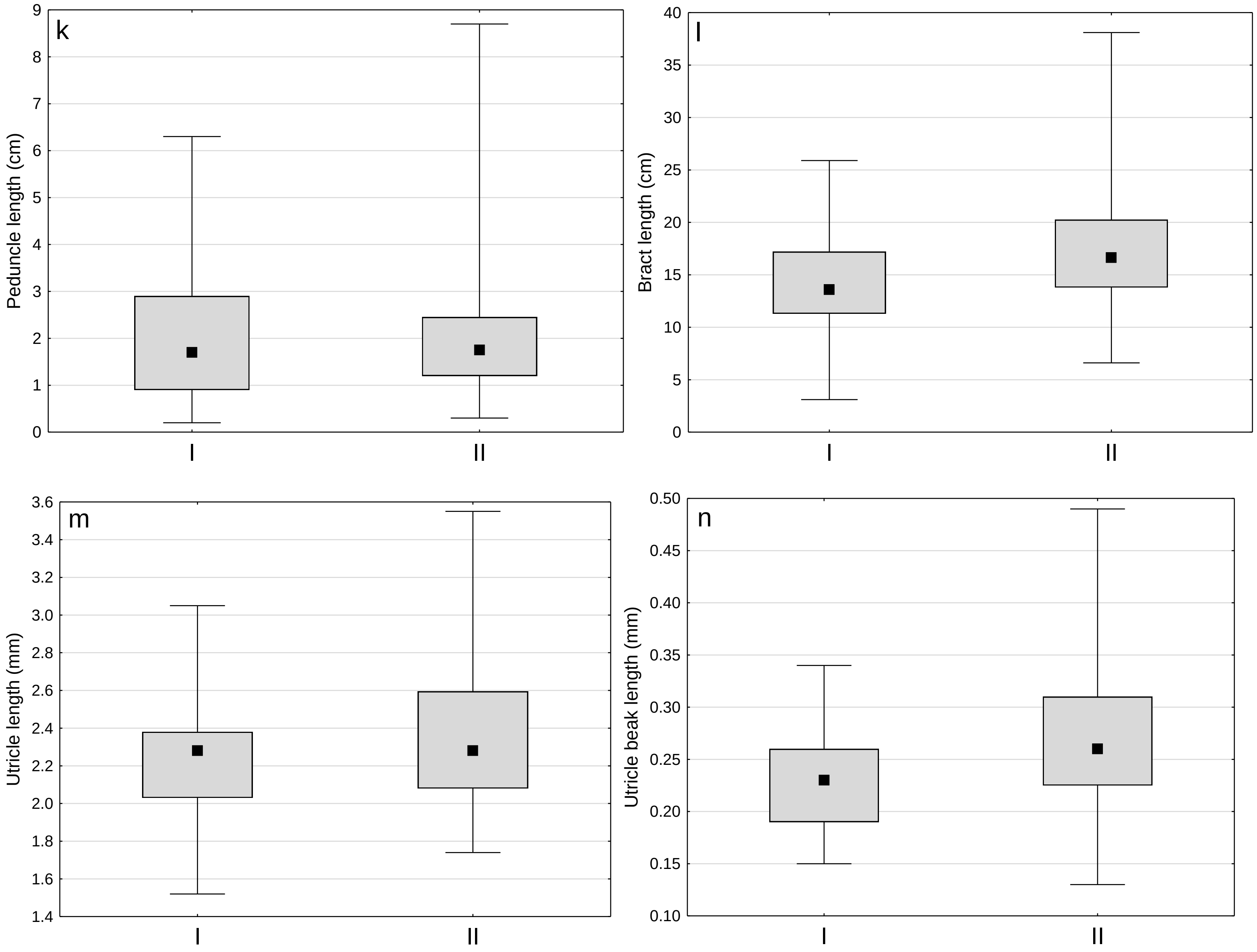

k
Peduncle length (cm)
0
1
2
3
4
5
6
7
8
9
I
II
l
Bract length (cm)
0
5
10
15
20
25
30
35
40
I
II
m
Utricle length (mm)
1.4
1.6
1.8
2.0
2.2
2.4
2.6
2.8
3.0
3.2
3.4
3.6
I
II
n
Utricle beak length (mm)
0.10
0.15
0.20
0.25
0.30
0.35
0.40
0.45
0.50
I
II

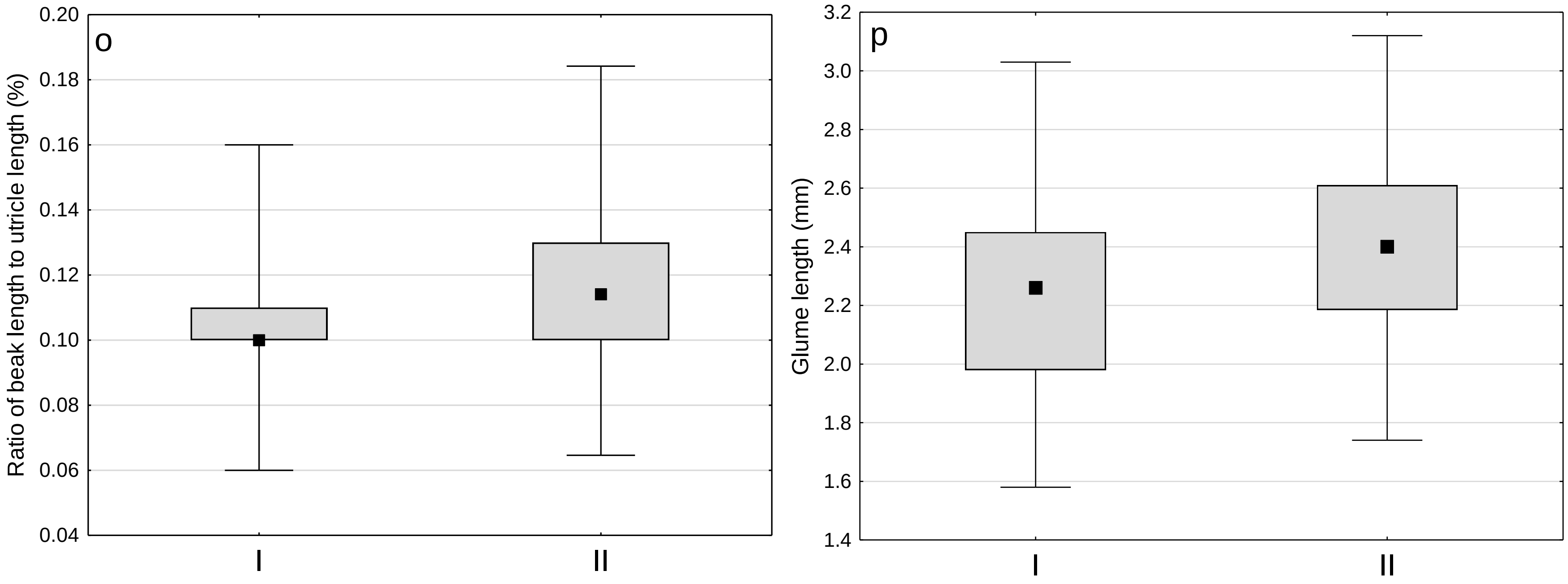
o
Ratio of beak length to utricle length (%)
0.20
0.18
0.16
0.14
0.12
0.10
0.08
0.06
0.04
I
II
p
Glume length (mm)
3.2
3.0
2.8
2.6
2.4
2.2
2.0
1.8
1.6
1.4
I
II
